# The selective SIK2/3 inhibitor GLPG3970 exhibits antidepressant-like effects via the hippocampal SIK2-CRTC1-CREB-BDNF pathway in chronic stress models

**DOI:** 10.3389/fphar.2026.1895911

**Published:** 2026-10-01

**Authors:** Jia-Yi Shen, Lu Chen, Fei-Yang Jin, Jun-Jie Qian, Feng Zhang, Ruo-Jia Wang, Meng-Jing Qi, Bo Jiang, Hua Fan

**Affiliations:** 1 Department of Pharmacology, School of Pharmacy, Nantong University, Nantong, Jiangsu, China; 2 Office of Research and Innovation, The First Affiliated Hospital, College of Clinical Medicine of Henan University of Science and Technology, Luoyang, Henan, China

**Keywords:** brain-derived neurotrophic factor, CREB-regulated transcription coactivator 1, cyclic AMP response element binding protein, depression, GLPG3970, salt-inducible kinase 2

## Abstract

**Introduction:**

Depression is a common mental disorder driven by biopsychosocial factors. Although traditional antidepressants serve as first-line therapies, they face clinical challenges, underscoring the need for novel therapeutic agents with improved efficacy. Our previous studies have identified hippocampal SIK2 as a key regulator in the pathogenesis of depression, acting through the cAMP response element-binding protein (CREB)-regulated transcription coactivator 1 (CRTC1)-CREB-brain derived neurotrophic factor (BDNF) signaling pathway. In this study, we investigated the antidepressant-like effects of GLPG3970, a novel selective inhibitor of SIK2/3, in male C57BL/6J mice.

**Methods:**

Chronic social defeat stress (CSDS) and chronic unpredictable mild stress (CUMS) models of depression were employed, combined with behavioral tests, virus-mediated gene transfer, Western blotting, co-immunoprecipitation, and immunofluorescence.

**Results:**

It was found that repeated administration of GLPG3970 significantly suppressed depressive-like behaviors in both CSDS and CUMS model mice, blocked stress-induced increases in hippocampal SIK2 expression and CRTC1 cytoplasmic translocation, and consequently restored the hippocampal BDNF signaling cascade and neurogenesis. More importantly, by using adeno-associated virus (AAV)-mediated overexpression of SIK2 or silencing of CRTC1/CREB/BDNF in the hippocampus, the antidepressant-like effects of GLPG3970 in mice were notably eliminated.

**Discussion:**

Collectively, these findings together demonstrate that the antidepressant-like actions of GLPG3970 in mice depend on the hippocampal SIK2-CRTC1-CREB-BDNF signaling pathway, highlighting its potential as a novel candidate for antidepressant therapy.

## Highlights

• GLPG3970 administration produced beneficial effects against chronic stress-induced depressive-like behaviors in mice.
• GLPG3970 treatment prevented the inhibitory effects of chronic stress on adult neurogenesis and the hippocampal BDNF signaling.
• The hippocampal SIK2-CRTC1-CREB-BDNF pathway is necessary for the therapeutic actions of GLPG3312 in chronic stress models.

## 1 Introduction

Major depressive disorder (MDD) is one of the most prevalent and debilitating mental health conditions worldwide, characterized by persistent low mood, anhedonia, and significant cognitive and social impairments (Monroe and Harkness, 2022; Thom et al., 2019). With over 264 million affected individuals globally and a high recurrence rate, MDD imposes substantial burdens on both individual health and public healthcare systems (Monroe and Harkness, 2022; Sheehan et al., 2017; Thom et al., 2019). Furthermore, depression is associated with increased risks of chronic comorbidities including type 2 diabetes, cardiovascular disease, and inflammatory bowel disease (Bisgaard et al., 2023; De Long et al., 2015; Rajan et al., 2020).

Based on the monoaminergic hypothesis, traditional antidepressants—including tricyclic antidepressants (TCAs), monoamine oxidase inhibitors (MAOIs), selective serotonin reuptake inhibitors (SSRIs), and serotonin-norepinephrine reuptake inhibitors (SNRIs)—have long been the mainstay of pharmacotherapy (Boku et al., 2018; López-Muñoz and Alamo, 2009). However, safety concerns, significant side effects, and a 30% treatment-resistant depression (TRD) rate underscore the urgent need for novel antidepressants with improved efficacy and tolerability (Boku et al., 2018; López-Muñoz and Alamo, 2009; Perez-Caballero et al., 2019).

The neuropathological mechanisms of depression are complex and incompletely understood, involving neurotransmitter abnormalities, hypothalamic–pituitary–adrenal (HPA) axis dysfunction, neuroinflammation, and impaired neuroplasticity (Dean and Keshavan, 2017; Fox and Lobo, 2019; Krishnan and Nestler, 2008; Massart et al., 2012; Ménard et al., 2016; Pastis et al., 2023). The hippocampus, a key brain region for memory and emotion, exhibits 5%–10% volume reduction in depressed patients, characterized by CA3 pyramidal cell degeneration, diminished dentate gyrus (DG) neurogenesis, and reduced synaptic plasticity (Bremner et al., 2000; Colla et al., 2007; Frodl et al., 2002).

In rodent models of depression, cAMP response element-binding protein (CREB) and brain-derived neurotrophic factor (BDNF) in the hippocampus are core mediators of stress adaptation and antidepressant effects (Dean and Keshavan, 2017; Krishnan and Nestler, 2008). The downstream molecules of BDNF include the membrane tyrosine kinase receptor B (TrkB) receptor and the extracellular signal-regulated kinase (ERK)/protein kinase B (AKT) pathway (Castrén and Monteggia, 2021). CREB directly regulates BDNF transcription, while CREB-regulated transcription coactivators (CRTCs) coactivate CREB through direct binding (Altarejos and Montminy, 2011; Takemori et al., 2007). CRTC activity is negatively regulated by salt-inducible kinases (SIKs), which phosphorylate CRTCs and promote their cytoplasmic retention (Darling and Cohen, 2021; Takemori et al., 2007). Our previous work demonstrated that chronic stress specifically upregulates hippocampal SIK2 (not SIK1 or SIK3), leading to CRTC1 phosphorylation, impaired nuclear translocation, reduced CRTC1-CREB complex formation, decreased BDNF biosynthesis, and consequent suppression of hippocampal neurogenesis and depression-like phenotypes (Jiang et al., 2019).

Targeted SIK2 inhibition has been shown to modulate the CRTC1–CREB–BDNF axis and ameliorate depression-like behaviors in mouse models (Huang et al., 2023; Liu et al., 2020). GLPG3970 is a newly developed selective SIK2/3 inhibitor with IC50 values of 7.8 nM for SIK2 and 3.8 nM for SIK3 (Peixoto et al., 2024). Although GLPG3970 inhibits both SIK2 and SIK3, chronic stress specifically modulates SIK2 in the hippocampus (Jiang et al., 2019). Given that the central pharmacological effects of GLPG3970 remain largely unexplored, this study investigated whether GLPG3970, via SIK2 inhibition, modulates the hippocampal CRTC1–CREB–BDNF pathway, reverses the suppression of hippocampal neurogenesis, and alleviates depressive-like symptoms in CSDS and CUMS model mice.

## 2 Materials and methods

### 2.1 Ethics statement

All animal experiments and related care procedures were conducted in accordance with the ARRIVE guidelines (Percie du Sert et al., 2020) and were approved by the Animal Welfare Committee of Nantong University. Animal welfare principles were consistently upheld throughout the study, and all efforts were made to minimize pain and discomfort to the animals.

### 2.2 Animals

Male C57BL/6J mice (8 weeks old) were used to establish depression models. CD1 mice (50 weeks old, male) were used as residents in the chronic social defeat stress (CSDS) paradigm and as intruders in the social interaction test. All mice were purchased from SLAC Laboratory Animal Company (Shanghai, China). Animals were housed under standard conditions: temperature maintained at 22 °C ± 2 °C, relative humidity at 50% ± 10%, and a 12 h light/dark cycle, with *ad libitum* access to food and water. To ensure animal welfare, water bottles and feed were regularly replaced, and bedding was changed weekly. Prior to experimentation, all mice underwent a one-week acclimation period. Sample sizes were determined by power analysis and also according to numerous previous studies (Hua et al., 2021; Huang et al., 2020; Jiang et al., 2022; Shi et al., 2022; Su et al., 2019; Wu et al., 2020; Zeng et al., 2020; Zhang et al., 2020; Zhao J. et al., 2020; Zhao X. et al., 2020), and mice were then randomly assigned to weight-matched experimental groups using a computer-generated randomization sequence. All behavioral tests were conducted between 8:00 a.m. and 5:00 p.m. and by investigators blinded to group allocation. Mice used for *in vitro* analyses were euthanized by cervical dislocation following carbon dioxide (CO_2_) anesthesia at 9:00 a.m., in accordance with standardized experimental protocols. The CO_2_ was delivered into the euthanasia chamber at a flow rate sufficient to displace 30% of the chamber volume per minute (e.g., 3 L/min for a 10 L chamber). After respiratory arrest, the CO_2_ flow was maintained for an additional 3 min before confirmatory cervical dislocation. *In vitro* data analysis was performed in a blinded manner using coded sample identities.

### 2.3 Drugs

Fluoxetine hydrochloride and GLPG3970 used in this study were purchased from TargetMol (Boston, MA, United States). The vehicle for both compounds consisted of 90% sodium chloride, 2% dimethyl sulfoxide (DMSO), and 8% Cremophor EL. All drugs were administered via intraperitoneal (i.p.) injection at a uniform volume of 10 mL/kg body weight. Dosing regimens (GLPG3970: 10 and 20 mg/kg; fluoxetine: 20 mg/kg) were determined based on published literature (Peixoto et al., 2024; Peng et al., 2023) and previous studies from our group (Jiang et al., 2019; Liu et al., 2020). Control animals received an equal volume of vehicle by intraperitoneal injection.

### 2.4 Chronic social defeat stress (CSDS)

The CSDS depression model was established according to previously described protocols (Huang et al., 2023; Jiang et al., 2019; Liu et al., 2020). Highly aggressive male CD1 breeder mice were selected as resident aggressors, and male C57BL/6J mice served as the experimental subjects. Each C57BL/6J mouse underwent a 10-min direct social confrontation with a novel CD1 aggressor mouse for 10 consecutive days, until the C57BL/6J mouse exhibited submissive postures within the 10-min session. After each daily session, the mice were separated by a perforated transparent barrier, which maintained visual and olfactory threat exposure for the test mouse. Following the 10-day defeat period, the defeated C57BL/6J mice were housed individually and treated with vehicle, fluoxetine, or GLPG3970 for 2 weeks. The negative control group was maintained under standard housing conditions without any intervention. Depressive-like behaviors were assessed using the forced swim test (FST), tail suspension test (TST), sucrose preference test (SPT), and social interaction test. Subsequently, biochemical analyses were performed on the C57BL/6J mice.

### 2.5 Chronic unpredictable mild stress (CUMS)

The CUMS depression model was established according to previously described protocols (Huang et al., 2023; Jiang et al., 2019; Liu et al., 2020). This model simulates uncontrollable and irregular chronic stressors encountered in human life by subjecting mice to long-term, randomized, mild stressors. In brief, C57BL/6J mice in the model group received one to two random and irregular stimuli per day for eight consecutive weeks. During the final 2 weeks of the CUMS procedure, mice in the treatment groups received daily i.p. injections of vehicle, fluoxetine, or GLPG3970. The specific stressors included water deprivation (23 h), food deprivation (23 h), physical restraint (2 h), cage rotation (30 min), light/dark cycle inversion, empty cage with 45 °C tilting (12 h), damp sawdust (12 h), and cold exposure (4 °C for 1 h). After the 8-week period, depressive-like behaviors were assessed using the FST, TST, and SPT. Afterwards, biochemical analyses were performed on the C57BL/6J mice.

### 2.6 FST

The FST protocol in this study was consistent with our previous reports (Huang et al., 2023; Jiang et al., 2019; Liu et al., 2020). C57BL/6J mice were placed individually into one transparent cylinder (45 cm height, 20 cm diameter) filled with fresh water (25 °C ± 1 °C, 15 cm depth) and forced to swim. The test lasted for 6 min, and the cumulative immobility time during the final 4 min was used as an indicator of depressive-like behavior. Immobility was defined as the mouse floating motionless on the water surface, making only slight movements necessary for breathing without active struggling. The experimenter was blinded to the group allocation and remained silent throughout the testing procedure.

### 2.7 TST

The TST protocol in this study was consistent with our previous reports (Huang et al., 2023; Jiang et al., 2019; Liu et al., 2020). In brief, the tip of each mouse’s tail was secured to a horizontal bar (65 cm above the floor) using adhesive tape, positioning the mouse in an inverted posture without contact with any other objects to prevent climbing. The duration of immobility was recorded over a 6-min period as an indicator of depressive-like behavior. Immobility was defined as a state of complete physical stillness. The testing procedure was conducted in silence, and the experimenters were blinded to the group allocation of the mice.

### 2.8 SPT

The SPT protocol used in this study was consistent with our previous reports (Huang et al., 2023; Jiang et al., 2019; Liu et al., 2020). Briefly, C57BL/6J mice were initially given free access to one bottle of pure water and one bottle of 1% (w/v) sucrose solution, along with adequate food, for the first 48 h. The positions of the two bottles were exchanged every 6 h to avoid location preference. Thereafter, both pure water and sucrose solution were removed, and mice were subjected to 18 h of fluid and food deprivation. Subsequently, during the 6-h test session, mice were re-exposed to one bottle of pure water and one bottle of 1% sucrose solution, with bottle positions switched every 2 h. The sucrose preference index was calculated as the volume of sucrose solution consumed divided by the total volume of liquid consumed, expressed as a percentage.

### 2.9 Social interaction test

The social interaction test protocol used in this study was consistent with our previous reports (Huang et al., 2023; Jiang et al., 2019; Liu et al., 2020). The experimental apparatus consisted of an open rectangular arena (50 × 50 × 45 cm), which served as the free-roaming area for the mice. A circular wire mesh cage was placed on one side of the arena to define the social interaction zone. The test consisted of two phases. During the first phase, each C57BL/6J mouse was allowed to freely explore the open arena alone for 5 minutes. In the second phase, the same mouse was reintroduced into the arena, and an unfamiliar CD1 male mouse was placed inside the wire mesh cage to serve as a social stimulus. The duration of time the experimental mouse spent in the social interaction area (defined as a 10-cm perimeter surrounding the wire cage) was recorded via a camera, and this measure was used to assess the extent of social avoidance behavior. To maintain environmental stability, the apparatus was cleaned with 20% ethanol after each test.

### 2.10 Open field test (OFT)

This method was used to assay the spontaneous locomotor activity of mice (Huang et al., 2023; Jiang et al., 2019; Liu et al., 2020). The OFT apparatus consisted of a 100 × 100 cm open arena with 50 cm high walls, and its floor was divided into 25 equal areas (20 × 20 cm). In brief, the mice were individually placed into the center of the apparatus, and behavior was observed for 5 min. The squares that each mouse crossed during the 5 min period were recorded. The apparatus was illuminated with a red bulb (50 W) and thoroughly cleaned after each trial.

### 2.11 Stereotaxic infusion and adeno-associated virus (AAV)-Mediated gene transfer

The experimental procedures involving adeno-associated virus (AAV) vectors were performed according to methods established by our laboratory (Jiang et al., 2019), using various AAV vectors expressing EGFP (AAV-Control-EGFP, AAV-SIK2-EGFP, AAV-Control-short hairpin RNA (shRNA)-EGFP, AAV-CRTC1-shRNA-EGFP, AAV-CREB-shRNA-EGFP, and AAV-BDNF-shRNA-EGFP; GeneChem, Shanghai, China), with the following shRNA target sequences: non-targeting control, 5′-TTC​TCC​GAA​CGT​GTC​ACG​T-3’; CRTC1, 5′-GCAGTTCAAC.

ATGATGGAGAA-3’; CREB, 5′-CAG​CAG​CTC​ATG​CAA​CAT​CAT-3’; and BDNF, 5′-TGA​GCG​TGT​GTG​ACA​GTA​TTA-3’ (Jiang et al., 2019). All viral vectors were diluted to 5 × 10^12^ genome copies/mL before use. In brief, C57BL/6J mice were anesthetized via i.p. injection of sodium pentobarbital (0.5%, 50 mg/kg body weight), and anesthesia was confirmed by the absence of corneal reflexes and the lack of response to painful stimuli. After fixing the mouse’s head, the scalp was disinfected sequentially with 75% ethanol and 1% hydrogen peroxide, followed by a midline incision and marking of the hippocampal target coordinates (AP = −2.3 mm, ML = ±1.6 mm, DV = +1.8 mm relative to bregma) (Jiang et al., 2019). Small burr holes were drilled bilaterally through the skull, and dual-guide cannulas (Plastics One, Roanoke, VA, United States) were implanted into the hippocampus and secured with dental cement, with each cannula fitted with an inserted dummy cannula and a dust cap. Viral infusion was performed using a microinjector (Hamilton, Fisher Labosi, France) at a flow rate of 0.2 μL/min (1 μL per hemisphere), after which the microinjector was left in place for 5 min to ensure adequate viral spread. Following a 14-day incubation period to achieve sufficient viral transduction throughout the hippocampal parenchyma, subsequent experiments were conducted.

### 2.12 Western blotting

Following behavioral assessments, the experimental C57BL/6J mice were euthanized, and their whole brains were rapidly removed for hippocampal tissue isolation according to established protocols (Huang et al., 2023; Jiang et al., 2019; Liu et al., 2020). The hippocampal tissues were homogenized in NP-40 lysis buffer (Beyotime Biotechnology, Shanghai, China) containing 1 mM PMSF, protease inhibitors, and phosphatase inhibitors using a homogenizer at 4 °C. The lysates were then centrifuged at 12,000 × g for 15 min at 4 °C to obtain the protein supernatant. An aliquot of the supernatant was mixed with 5 × sodium dodecyl sulfate (SDS) loading buffer, and the remaining supernatant was used for protein concentration determination using the BCA method (Beyotime Biotechnology). For immunoblotting, equal amounts of protein from each group were separated by electrophoresis on 10% or 12% SDS-polyacrylamide gels and transferred onto nitrocellulose membranes. The membranes were then blocked with 5% non-fat milk in Tris-buffered saline containing 0.1% Tween-20 (TBST) for 2 h at room temperature, followed by overnight incubation at 4 °C with primary antibodies with gentle agitation. The following primary antibodies were used: anti-SIK2 (Cell Signaling Technology [CST], Danver, United States; 6,919, 1:1,000), anti-CRTC1 (CST, 2,587, 1:1,000), anti-phospho-CRTC1 (Merck Millipore, ABE560, 1:500), anti-BDNF (Abcam, Bristol, UK; ab108319, 1:300), anti-TrkB (Thermo Fisher, Waltham, MA, United States; PA5-120787, 1:1,000), anti-phospho-TrkB (Thermo Fisher; PA5-36695; 1:500), anti-ERK1/2 (CST, 9,102, 1:1,000), anti-phospho-ERK1/2 (CST, 9,101, 1:500), anti-AKT (CST, 9,272, 1:1,000), anti-phospho-AKT (CST, 4,060, 1:500), anti-CREB (CST, 9,197, 1:1,000), anti-phospho-CREB (CST, 9,198, 1:500), anti-Histone H3 (CST, 9,715, 1:500), and anti-β-actin (Abcam, ab8227, 1:2000). After washing three times with TBST (10 min each), the membranes were incubated with appropriate IRDye 680-conjugated secondary antibodies (1:5,000 dilution) for 2 h at room temperature protected from light. Protein bands were visualized and quantified using the Odyssey CLx infrared imaging system (LI-COR Biosciences, Lincoln, NE, United States).

For cytoplasmic and nuclear fractionation, proteins were extracted using the NE-PER™ Nuclear and Cytoplasmic Extraction Kit (78833; Thermo Fisher) according to the manufacturer’s instructions.

### 2.13 Co-immunoprecipitation (Co-IP)

Following euthanasia of C57BL/6J mice, the hippocampal tissues were rapidly isolated on ice and homogenized at 4 °C in NP-40 lysis buffer containing 1 mM PMSF, protease inhibitors, and phosphatase inhibitors using a homogenizer. The homogenate was centrifuged at 12,000 × g for 15 min at 4 °C to obtain the supernatant, and protein concentrations were quantified using a BCA kit (Beyotime Biotechnology). The procedures for detecting CRTC1-CREB protein binding in the hippocampus were performed as previously described (Huang et al., 2023; Jiang et al., 2019; Liu et al., 2020). Briefly, aliquots of the supernatant containing 500 μg of protein were incubated with 5 μg of anti-CRTC1 (CST, 2,587) or anti-CREB (CST, 9,197) antibody on a shaking incubator at 4 °C, with the remaining untreated supernatant retained as an input control. Protein A/G Plus Agarose beads, pre-washed twice with standard wash buffer, were added to the reaction mixture and incubated at room temperature for 2 h. The reaction mixture was then washed five times with high-salt washing buffer and centrifuged at 3,000 × g for 3 min at 4 °C to collect the bead-bound protein pellet. The pelleted beads were resuspended in SDS loading buffer and boiled at 95 °C for 5 min to elute the immune complexes. Finally, the eluted proteins were analyzed by Western blotting using (CST, 2,587) or anti-CREB (CST, 9,197) antibody.

### 2.14 Immunofluorescence (IF)

C57BL/6J mice were anesthetized with 0.5% sodium pentobarbital (50 mg/kg, i.p.) and sequentially perfused with physiological saline followed by 4% paraformaldehyde (PFA) diluted in 0.1 M phosphate-buffered saline (PBS). Whole brains were removed and post-fixed in 4% PFA overnight at 4 °C, then dehydrated in 30% sucrose in 0.1 M PBS for 2 days at 4 °C. Coronal hippocampal slices (25 μm) were serially sectioned using a cryostat microtome (Leica, Wetzlar, Germany) and stored in 0.1 M PBS at 4 °C. For the DG subregion of the hippocampus, doublecortin (DCX) and 4′, 6-diamidino-2′-phenylindole (DAPI) staining was performed. The following reagents were used: rabbit primary anti-DCX antibody (1:100; CST, 4,604), fluorescein isothiocyanate (FITC)-conjugated goat anti-rabbit secondary antibody (1:50; Thermo Fisher), and DAPI staining solution (Beyotime Biotechnology). The experimental procedures and quantification methods have been described in detail previously (Huang et al., 2023; Jiang et al., 2019; Liu et al., 2020). Briefly, brain sections were sequentially incubated with 0.3% Triton X-100% and 3% bovine serum albumin (BSA) for 30 min each, followed by overnight incubation at 4 °C with the diluted primary anti-DCX antibody. After washing with PBS, sections were incubated with diluted FITC-labeled secondary antibody for 2 h, then with DAPI for 10 min. Following coverslipping, quantitative analysis was performed using a confocal laser scanning system (Leica Microsystems, Wetzlar, Germany). For DCX-labeled (DCX^+^) cells, every sixth serial hippocampal section (spaced 150 μm apart) covering the bilateral rostrocaudal DG region (AP: −1.22 mm to −2.80 mm relative to bregma) was selected, and all DCX^+^ cells within the granule cell layer (GCL) and subgranular zone (SGZ) were counted.

For hippocampal neuronal nuclei (NeuN)/5-Bromo-2′-deoxyuridine (Brdu) co-labeling, the test mice were injected with Brdu (4 × 75 mg/kg at 2-h intervals/d) for 2 days, then anesthetized and perfused after 28 days. After post-fixing and dehydration, 25 µm coronal sections were collected and treated as follows: 1, incubation in 50% formamide/2 × SSC at 65 °C for 2 h; 2, incubation in 2 N HCl at 37 °C for 30 min; 3, incubation in 0.1 M boric acid buffer (pH 8.5) at room temperature for 10 min; 4, incubation in 0.3% Triton X-100 for 30 min; 5, incubation in 3% BSA for 30 min; 6, incubation with mouse anti-Brdu (2 μg/mL, Roche, Basel, Switzerland) and rabbit anti-NeuN (1:500; Abcam) antibodies overnight at 4 °C; 7, washing; 8, incubation with FITC-conjugated anti-rabbit and rhodamine-conjugated anti-mouse secondary antibodies (1:50; Thermo Fisher) at room temperature for 2 h; 9, washing; 10, coverslipping and observation. The sections were visualized using a DM6B fluorescence microscope (Leica Microsystems). Examinations of the NeuN^+^/BrdU^+^ co-labelling cells were confined to DG. Quantifications of the NeuN^+^/BrdU^+^ cells were respectively conducted from 1-in-6 series of hippocampal sections spaced at 150 μm and spanning the rostrocaudal region of DG bilaterally. Every NeuN^+^/BrdU^+^ cell within GCL and SGZ was counted.

### 2.15 Statistical analysis

Statistical analyses were performed using the SPSS 26.0 software (SPSS Inc, Chicago, United States). All data are presented as the mean ± standard error of the mean (SEM). Prior to statistical analyses, all datasets were tested for normality using the Shapiro-Wilk test and for homogeneity of variances using Levene’s test. All data met the assumptions of normality and homogeneity of variances for parametric testing. After confirming homogeneity of variances, two-way analysis of variance (ANOVA) was used to compare means across multiple groups, followed by *post hoc* pairwise comparisons with Bonferroni correction. One-way ANOVA followed by Tukey’s *post hoc* test was applied where appropriate. A significance threshold of *P* < 0.05 was considered statistically significant.

## 3 Results

### 3.1 Repeated injection of GLPG3970 significantly prevents CSDS- and CUMS-Induced depressive-like behaviors and reverses SIK2-CRTC1 system dysfunction in the hippocampus

To investigate whether GLPG3970 treatment exerts antidepressant-like effects, we employed two well-established and widely used depression models, CSDS (Figure 1A) and CUMS (Figure 2A), with fluoxetine serving as the positive control drug. As shown in Figures 1A, 2A, compared with the control group, mice exposed to CSDS or CUMS exhibited increased immobility duration in the FST and TST (helplessness and despair behavior) and reduced sucrose preference (anhedonia), demonstrating marked depressive-like behavior (n = 10). Furthermore, CSDS-induced mice displayed lower frequency of social interaction with CD1 intruders than control mice (n = 10; Figure 1A). Compared with vehicle-treated stress model mice, i.p. administration of GLPG3970 at 10 mg/kg or 20 mg/kg significantly reduced immobility time of CSDS and CUMS model mice in both the FST [ANOVA for CSDS: CSDS, F(1, 72) = 21.744, *P* < 0.01; Drug treatment, F(3, 72) = 18.579, *P* < 0.01; Interaction, F(3, 72) = 14.207, *P* < 0.01. ANOVA for CUMS: CUMS, F(1, 72) = 24.022, *P* < 0.01; Drug treatment, F(3, 72) = 16.965, *P* < 0.01; Interaction, F(3, 72) = 12.833, *P* < 0.01] and TST [ANOVA for CSDS: CSDS, F(1, 72) = 23.066, *P* < 0.01; Drug treatment, F(3, 72) = 19.164, *P* < 0.01; Interaction, F(3, 72) = 13.449, *P* < 0.01. ANOVA for CUMS: CUMS, F(1, 72) = 26.559, *P* < 0.01; Drug treatment, F(3, 72) = 22.305, *P* < 0.01; Interaction, F(3, 72) = 17.482, *P* < 0.01], while markedly increasing sucrose preference [ANOVA for CSDS: CSDS, F(1, 72) = 25.011, *P* < 0.01; Drug treatment, F(3, 72) = 20.662, *P* < 0.01; Interaction, F(3, 72) = 15.432, *P* < 0.01. ANOVA for CUMS: CUMS, F(1, 72) = 19.106, *P* < 0.01; Drug treatment, F(3, 72) = 16.753, *P* < 0.01; Interaction, F(3, 72) = 11.048, *P* < 0.01] of CSDS and CUMS model mice (n = 10, *P <* 0.01; Figures 1A, 2A). Additionally, GLPG3970-treated CSDS model mice exhibited a significant increase in social interaction frequency with CD1 intruders [ANOVA: CSDS, F(1, 72) = 32.567, *P* < 0.01; Drug treatment, F(3, 72) = 26.703, *P* < 0.01; Interaction, F(3, 72) = 22.118, *P* < 0.01] compared with vehicle-injected stressed mice (n = 10, *P <* 0.01; Figure 1A). These behavioral alterations collectively indicate that GLPG3970 treatment produces significant antidepressant-like effects. Among the tested doses, GLPG3970 at 20 mg/kg demonstrated superior efficacy, comparable to that of fluoxetine at the same dose. Notably, GLPG3970 treatment exerted no significant effects on FST, TST, SPT, or social interaction test outcomes in naïve control mice (n = 10; Figures 1A, 2A). To exclude the possibility that the observed reductions in immobility time in the FST and TST were attributable to changes in locomotor activity, we performed the OFT. As shown in Supplementary Figures S1A, S1B, neither stress exposure nor drug treatment significantly altered peripheral activity, central activity, or total activity in mice (n = 10), confirming that the behavioral effects observed reflect genuine antidepressant-like actions rather than non-specific locomotor alterations.

**FIGURE 1:**
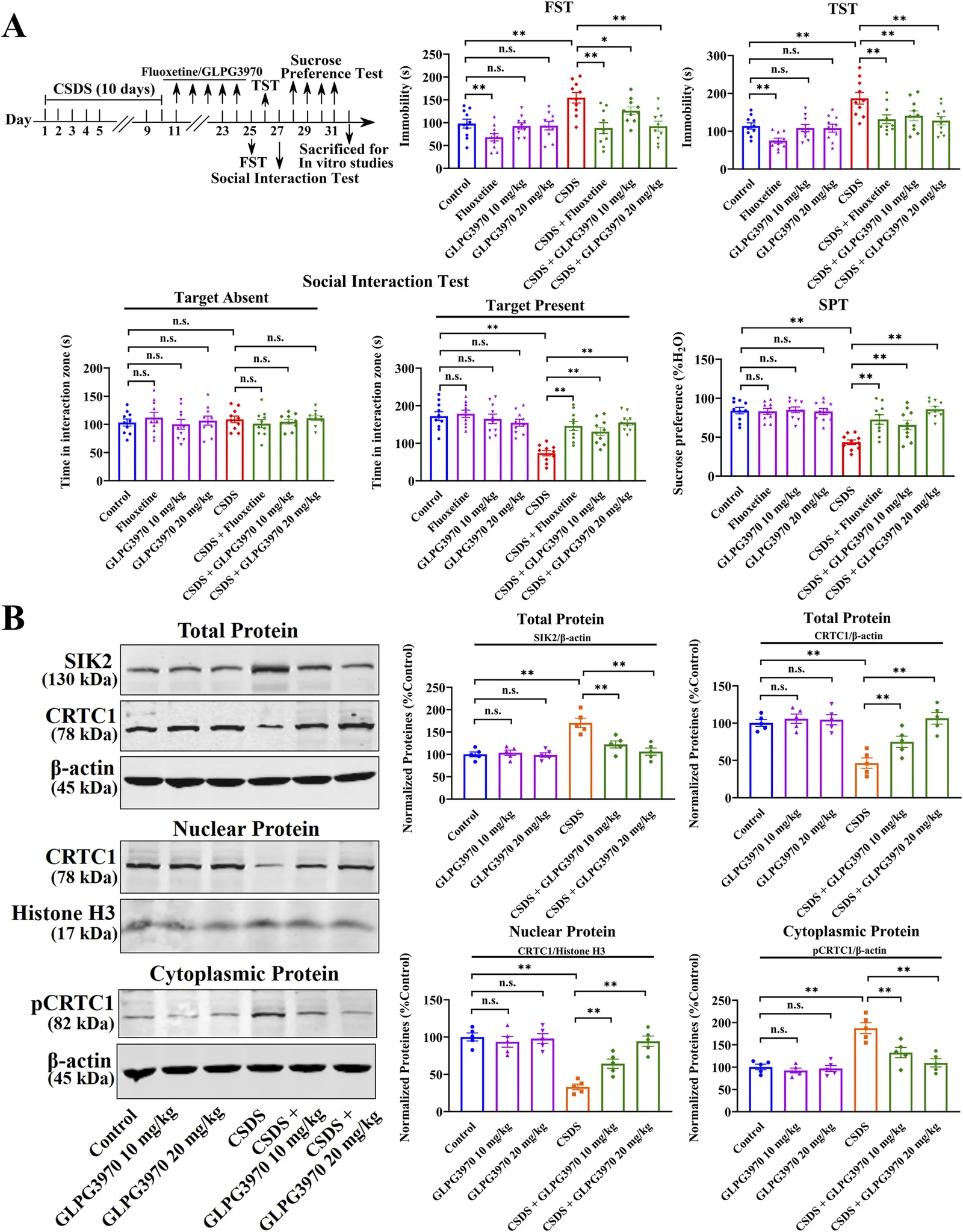
Depressive-like behaviors and abnormal alterations in the hippocampal SIK2-CRTC1 signaling pathway in mice exposed to CSDS were reversed by i.p. administration of GLPG3970. **(A)** The experimental procedure timeline and behavioral testing results. GLPG3970 administration reversed CSDS-induced depressive-like behaviors in mice, as evidenced by reduced immobility time in the FST and TST, increased frequency of social interaction as well as restored sucrose preference in the SPT. **(B)** Representative Western blot images (with molecular weight markers) and densitometric quantification of SIK2, total CRTC1, nuclear CRTC1, and cytoplasmic pCRTC1 expression in the hippocampus of mice. Notably, CSDS-induced alterations in the expression of these proteins were reversed by GLPG3970 treatment. All data are presented as means ± S.E.M. of *n* = 10 (for A) or 5 (for B) mice per group; *P < 0.05, **P < 0.01 by two-way ANOVA with Bonferroni correction; n.s., no significance.

**FIGURE 2:**
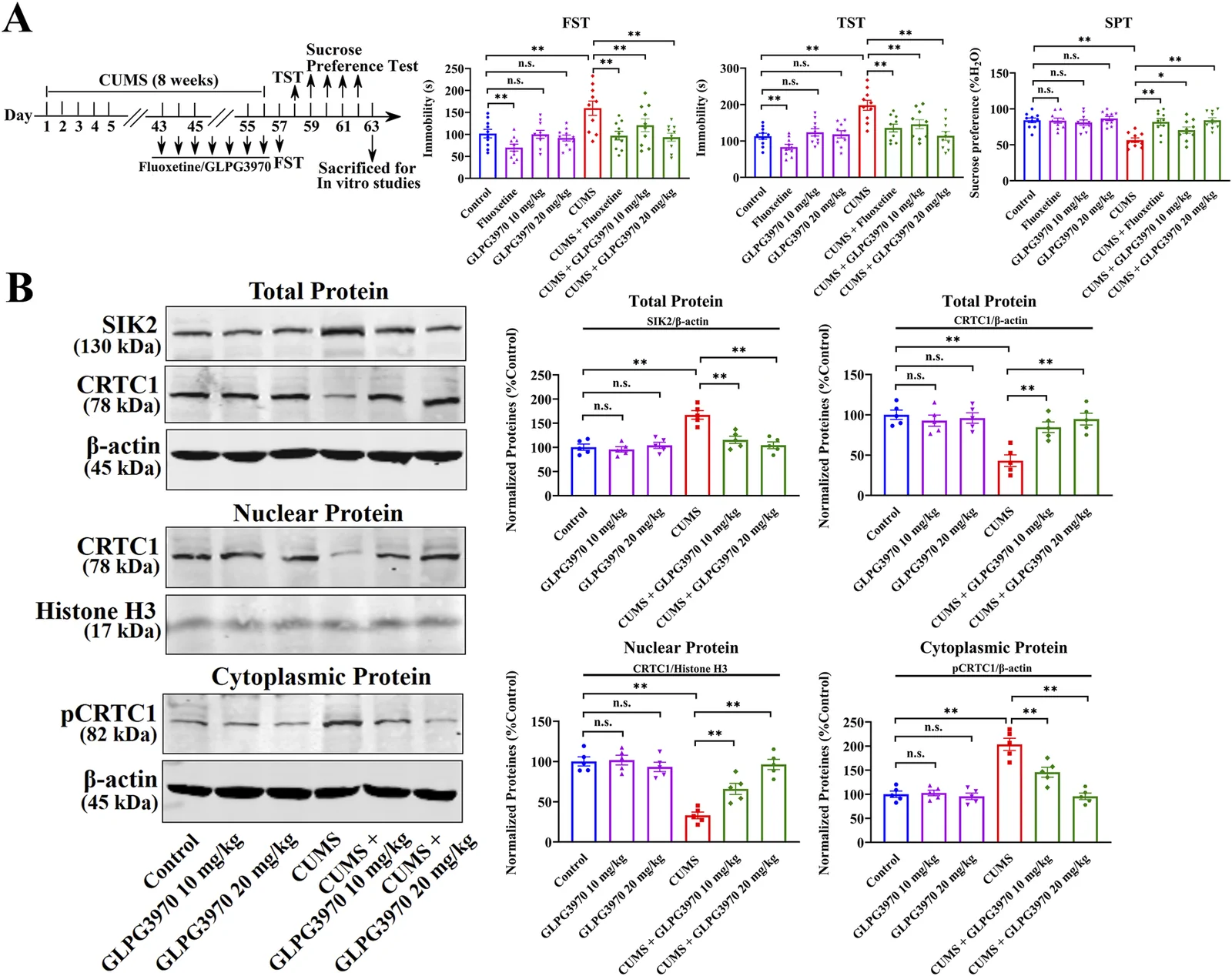
Depressive-like behaviors and abnormal alterations in the hippocampal SIK2-CRTC1 signaling pathway in mice exposed to CUMS were reversed by i.p. administration of GLPG3970. **(A)** The experimental procedure timeline and behavioral testing results. GLPG3970 administration also reversed CUMS-induced depressive-like behaviors in mice, as evidenced by reduced immobility time in the FST and TST, as well as restored sucrose preference in the SPT. **(B)** Representative Western blot images (with molecular weight markers) and densitometric quantification of SIK2, total CRTC1, nuclear CRTC1, and cytoplasmic pCRTC1 expression in the hippocampus of mice. Notably, CUMS-induced alterations in the expression of these proteins were reversed by GLPG3970 treatment. All data are presented as means ± S.E.M. of *n* = 10 (for A) or 5 (for B) mice per group; *P < 0.05, **P < 0.01 by two-way ANOVA with Bonferroni correction; n.s., no significance.

To examine alterations in the hippocampal SIK2-CRTC1 signaling system across groups, Western blotting and Co-IP were performed following behavioral testing. As shown in Figures 1B, 2B, 3A,B, the protein expression levels of SIK2 and cytoplasmic pCRTC1 were significantly upregulated in the hippocampus of CSDS-exposed and CUMS-exposed mice, whereas the levels of total CRTC1, nuclear CRTC1, and CRTC1-CREB binding were significantly downregulated (n = 5, *P <* 0.01), which is fully consistent with our previous report (Jiang et al., 2019). In contrast, i.p. injection of GLPG3970 significantly reversed these abnormal protein expression alterations. Statistical analysis revealed that compared with vehicle-treated stressed mice, those stressed mice receiving GLPG3970 at 10 mg/kg or 20 mg/kg exhibited reduced hippocampal SIK2 [ANOVA for CSDS: CSDS, F(1, 24) = 27.618, *P* < 0.01; Drug treatment, F(2, 24) = 23.446, *P* < 0.01; Interaction, F(2, 24) = 20.153, *P* < 0.01. ANOVA for CUMS: CUMS, F(1, 24) = 25.786, *P* < 0.01; Drug treatment, F(2, 24) = 22.341, *P* < 0.01; Interaction, F(2, 24) = 18.329, *P* < 0.01] and cytoplasmic pCRTC1 [ANOVA for CSDS: CSDS, F(1, 24) = 30.115, *P* < 0.01; Drug treatment, F(2, 24) = 25.299, *P* < 0.01; Interaction, F(2, 24) = 21.603, *P* < 0.01. ANOVA for CUMS: CUMS, F(1, 24) = 35.102, *P* < 0.01; Drug treatment, F(2, 24) = 30.667, *P* < 0.01; Interaction, F(2, 24) = 24.813, *P* < 0.01] protein expression levels (n = 5, *P <* 0.01, Figures 1B, 2B), whereas displayed increased levels of hippocampal total CRTC1 [ANOVA for CSDS: CSDS, F(1, 24) = 33.904, *P* < 0.01; Drug treatment, F(2, 24) = 27.188, *P* < 0.01; Interaction, F(2, 24) = 23.549, *P* < 0.01. ANOVA for CUMS: CUMS, F(1, 24) = 36.559, *P* < 0.01; Drug treatment, F(2, 24) = 31.428, *P* < 0.01; Interaction, F(2, 24) = 26.375, *P* < 0.01], nuclear CRTC1 [ANOVA for CSDS: CSDS, F(1, 24) = 45.175, *P* < 0.01; Drug treatment, F(2, 24) = 38.272, *P* < 0.01; Interaction, F(2, 24) = 29.147, *P* < 0.01. ANOVA for CUMS: CUMS, F(1, 24) = 41.926, *P* < 0.01; Drug treatment, F(2, 24) = 34.671, *P* < 0.01; Interaction, F(2, 24) = 28.305, *P* < 0.01], and CRTC1-CREB binding [ANOVA for CSDS and (CRTC1 from CREB binding): CSDS, F(1, 24) = 46.504, *P* < 0.01; Drug treatment, F(2, 24) = 38.169, *P* < 0.01; Interaction, F(2, 24) = 33.272, *P* < 0.01. ANOVA for CSDS and (CREB from CRTC1 binding): CSDS, F(1, 24) = 28.457, *P* < 0.01; Drug treatment, F(2, 24) = 24.198, *P* < 0.01; Interaction, F(2, 24) = 19.336, *P* < 0.01. ANOVA for CUMS and (CRTC1 from CREB binding): CUMS, F(1, 24) = 48.714, *P* < 0.01; Drug treatment, F(2, 24) = 41.088, *P* < 0.01; Interaction, F(2, 24) = 35.175, *P* < 0.01. ANOVA for CUMS and (CREB from CRTC1 binding): CUMS, F(1, 24) = 27.099, *P* < 0.01; Drug treatment, F(2, 24) = 23.103, *P* < 0.01; Interaction, F(2, 24) = 17.684, *P* < 0.01] (n = 5, *P <* 0.01; Figures 1B, 2B, 3A,B). Moreover, these biological effects were more pronounced at the higher dose of GLPG3970 (20 mg/kg). Furthermore, GLPG3970 treatment had no significant effects on the protein expression of the hippocampal SIK2-CRTC1 signaling system in naïve control mice (n = 5; Figures 1B, 2B, 3A,B).

**FIGURE 3:**
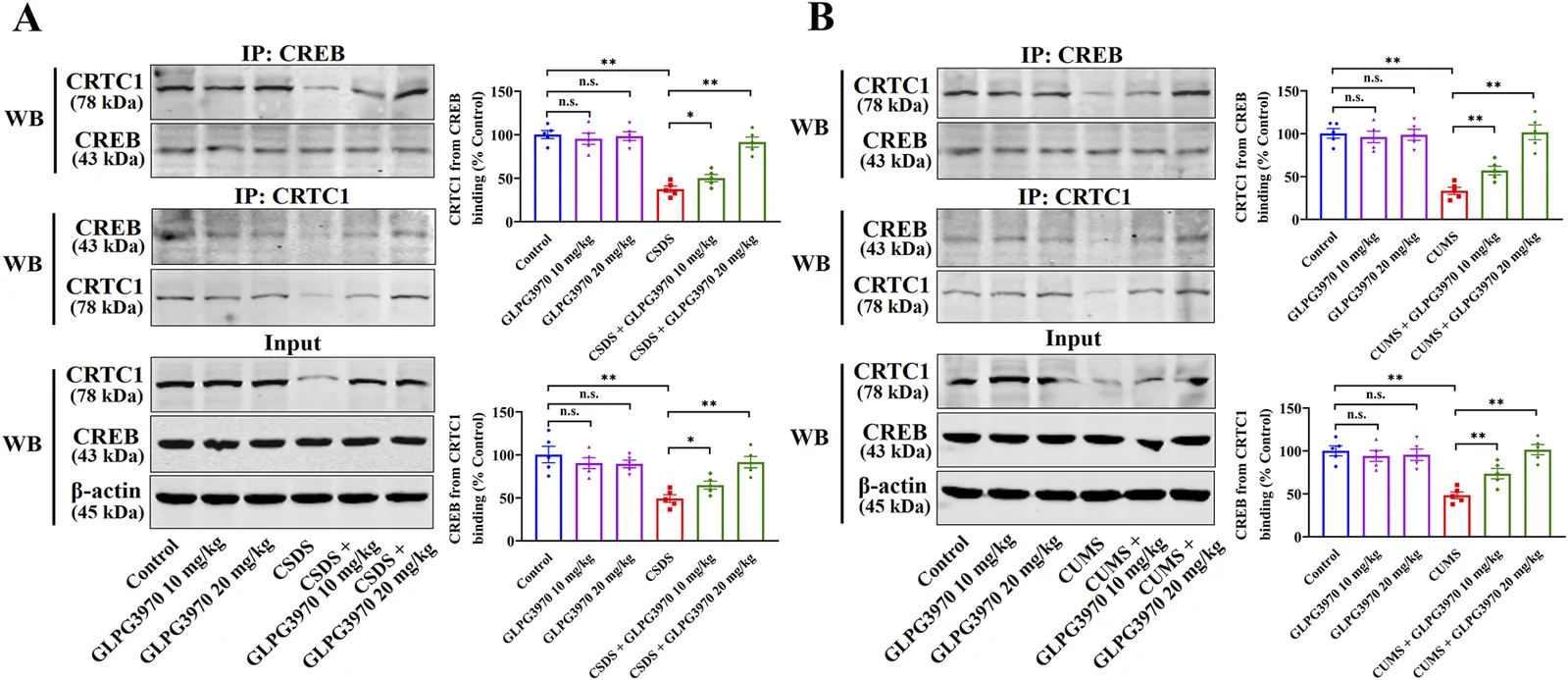
The diminished CRTC1-CREB binding levels in the hippocampus of mice subjected to CSDS or CUMS were reversed by i.p. administration of GLPG3970. **(A)** Representative Co-IP images (with molecular weight markers) and quantitative analysis results demonstrating the effects of CSDS and GLPG3970 on CRTC1-CREB protein binding in the hippocampus of mice. **(B)** Representative Co-IP images (with molecular weight markers) and quantitative analysis results demonstrating the effects of CUMS and GLPG3970 on CRTC1-CREB protein binding in the hippocampus of mice. All data are presented as means ± S.E.M. of *n* = 5 mice per group; *P < 0.05, **P < 0.01 by two-way ANOVA with Bonferroni correction; n.s., no significance.

Collectively, these findings demonstrate that repeated administration of GLPG3970 not only alleviates CSDS- and CUMS-induced depressive-like behaviors in mice but also normalizes the activity of the hippocampal SIK2-CRTC1 signaling pathway.

### 3.2 Repeated injection of GLPG3970 notably reverses the inhibitory effects of CSDS and CUMS on the hippocampal BDNF signaling pathway and adult neurogenesis

BDNF is one of the core molecules regulating hippocampal neurogenesis (Han et al., 2025). BDNF and its downstream signaling molecules are involved in the development and maintenance of neural plasticity through bidirectional regulatory mechanisms and are closely associated with the pathophysiological processes of depression (Björkholm and Monteggia, 2016; Castrén and Monteggia, 2021). Accordingly, this study examined changes in the expression of proteins related to the BDNF signaling pathway and adult neurogenesis across all experimental groups.

As shown in Figures 4–7, compared with the control group, CSDS-exposed and CUMS-exposed exhibited significantly downregulated expression levels of BDNF, pTrkB, pERK1/2, pAKT, and pCREB in hippocampal tissue, alongside markedly diminished adult neurogenesis (evidenced by reduced amounts of both DCX^+^ and NeuN^+^/BrdU^+^ cells in the DG) (n = 5, *P <* 0.01). This finding is consistent with the conclusions of our previous research (Jiang et al., 2019). Compared with vehicle-treated stressed mice, i.p. administration of GLPG3970 at 10 mg/kg or 20 mg/kg significantly upregulated the expression levels of BDNF [ANOVA for CSDS: CSDS, F(1, 24) = 37.674, *P* < 0.01; Drug treatment, F(2, 24) = 29.449, *P* < 0.01; Interaction, F(2, 24) = 22.525, *P* < 0.01. ANOVA for CUMS: CUMS, F(1, 24) = 38.196, *P* < 0.01; Drug treatment, F(2, 24) = 32.448, *P* < 0.01; Interaction, F(2, 24) = 27.505, *P* < 0.01], pTrkB [ANOVA for CSDS: CSDS, F(1, 24) = 36.697, *P* < 0.01; Drug treatment, F(2, 24) = 30.402, *P* < 0.01; Interaction, F(2, 24) = 25.313, *P* < 0.01. ANOVA for CUMS: CUMS, F(1, 24) = 27.485, *P* < 0.01; Drug treatment, F(2, 24) = 23.144, *P* < 0.01; Interaction, F(2, 24) = 18.062, *P* < 0.01], pERK1/2 [ANOVA for CSDS: CSDS, F(1, 24) = 26.889, *P* < 0.01; Drug treatment, F(2, 24) = 22.413, *P* < 0.01; Interaction, F(2, 24) = 18.256, *P* < 0.01. ANOVA for CUMS: CUMS, F(1, 24) = 18.647, *P* < 0.01; Drug treatment, F(2, 24) = 15.701, *P* < 0.01; Interaction, F(2, 24) = 11.469, *P* < 0.01], pAKT [ANOVA for CSDS: CSDS, F(1, 24) = 30.597, *P* < 0.01; Drug treatment, F(2, 24) = 25.034, *P* < 0.01; Interaction, F(2, 24) = 20.277, *P* < 0.01. ANOVA for CUMS: CUMS, F(1, 24) = 34.081, *P* < 0.01; Drug treatment, F(2, 24) = 29.306, *P* < 0.01; Interaction, F(2, 24) = 25.177, *P* < 0.01], and pCREB [ANOVA for CSDS: CSDS, F(1, 24) = 24.166, *P* < 0.01; Drug treatment, F(2, 24) = 19.058, *P* < 0.01; Interaction, F(2, 24) = 14.231, *P* < 0.01. ANOVA for CUMS: CUMS, F(1, 24) = 37.102, *P* < 0.01; Drug treatment, F(2, 24) = 31.634, *P* < 0.01; Interaction, F(2, 24) = 24.293, *P* < 0.01] in stressed mice, concurrently enhancing adult neurogenesis capacity [ANOVA for CSDS and DCX^+^: CSDS, F(1, 24) = 52.119, *P* < 0.01; Drug treatment, F(2, 24) = 47.326, *P* < 0.01; Interaction, F(2, 24) = 38.204, *P* < 0.01. ANOVA for CUMS and DCX^+^: CUMS, F(1, 24) = 55.408, *P* < 0.01; Drug treatment, F(2, 24) = 45.642, *P* < 0.01; Interaction, F(2, 24) = 36.057, *P* < 0.01. ANOVA for CSDS and NeuN^+^/BrdU^+^: CSDS, F(1, 24) = 48.194, *P* < 0.01; Drug treatment, F(2, 24) = 38.447, *P* < 0.01; Interaction, F(2, 24) = 27.816, *P* < 0.01. ANOVA for CUMS and NeuN^+^/BrdU^+^: CUMS, F(1, 24) = 45.204, *P* < 0.01; Drug treatment, F(2, 24) = 36.315, *P* < 0.01; Interaction, F(2, 24) = 24.736, *P* < 0.01] (n = 5, *P <* 0.01; Figures 4–7). Statistical analyses demonstrated that the regulatory efficacy of GLPG3970 at the dose of 20 mg/kg was more pronounced. Notably, no significant differences were observed in the expression levels of total TrkB, ERK1/2, AKT, and CREB proteins in the hippocampal tissues of mice across all experimental groups (n = 5). These findings indicate that repeated i.p. administration of GLPG3970 effectively reversed the inhibitory effects of CSDS and CUMS on the BDNF signaling pathway and adult neurogenesis in the mouse hippocampus.

**FIGURE 4:**
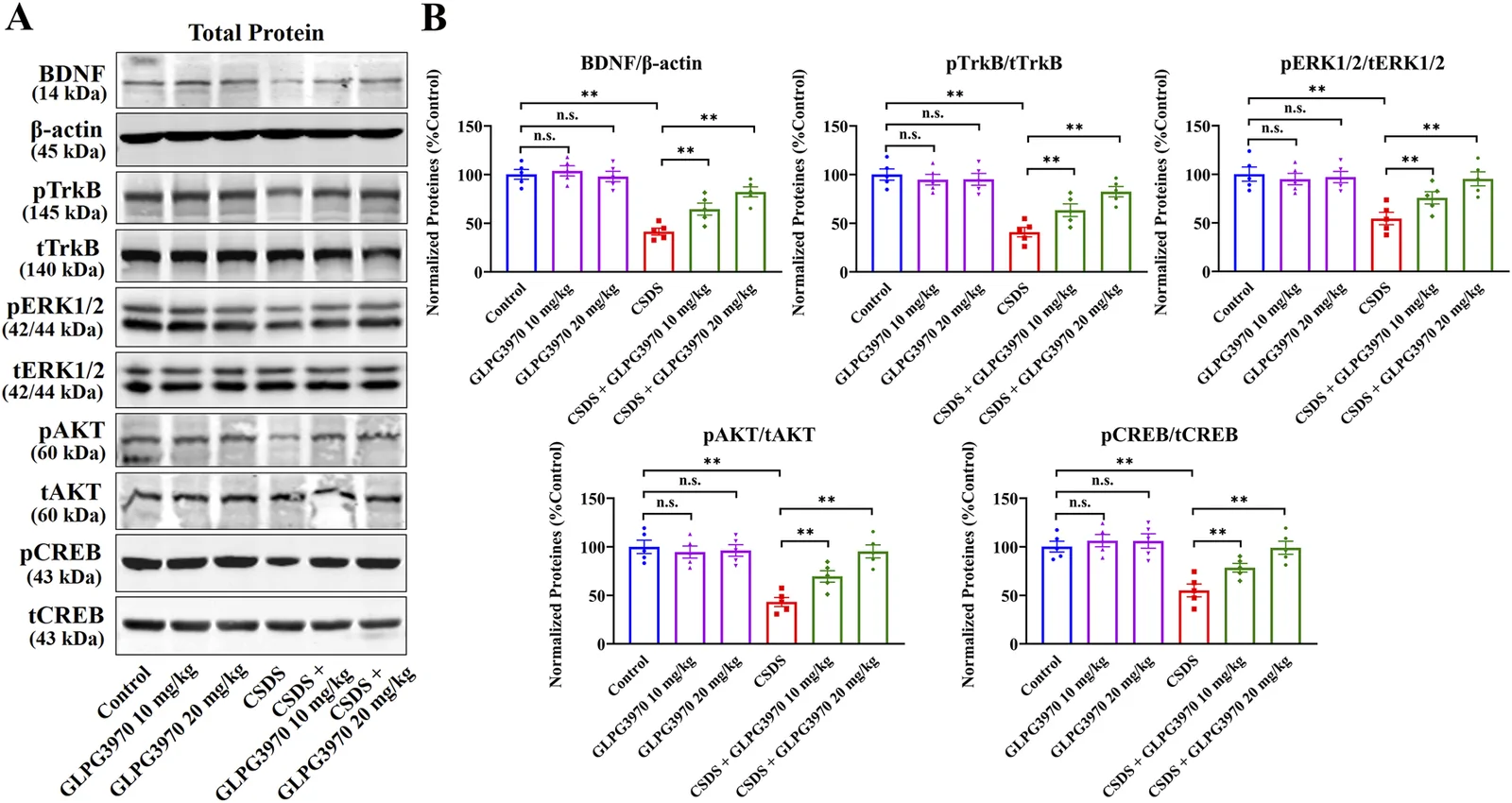
The reduction in hippocampal BDNF signaling in mice exposed to CSDS was reversed by i.p. administration of GLPG3970. **(A)** Representative Western blot images (with molecular weight markers). **(B)** Quantitative analyses showing the effects of CSDS and GLPG3970 on the protein levels of BDNF, pTrkB, pERK1/2, pAKT, pCREB, β-actin, total TrkB, total ERK1/2, total AKT, and total CREB in the hippocampus of mice. All results were presented as means ± S.E.M. of *n* = 5 mice per group; **P < 0.01 by two-way ANOVA with Bonferroni correction; n.s., no significance.

**FIGURE 5:**
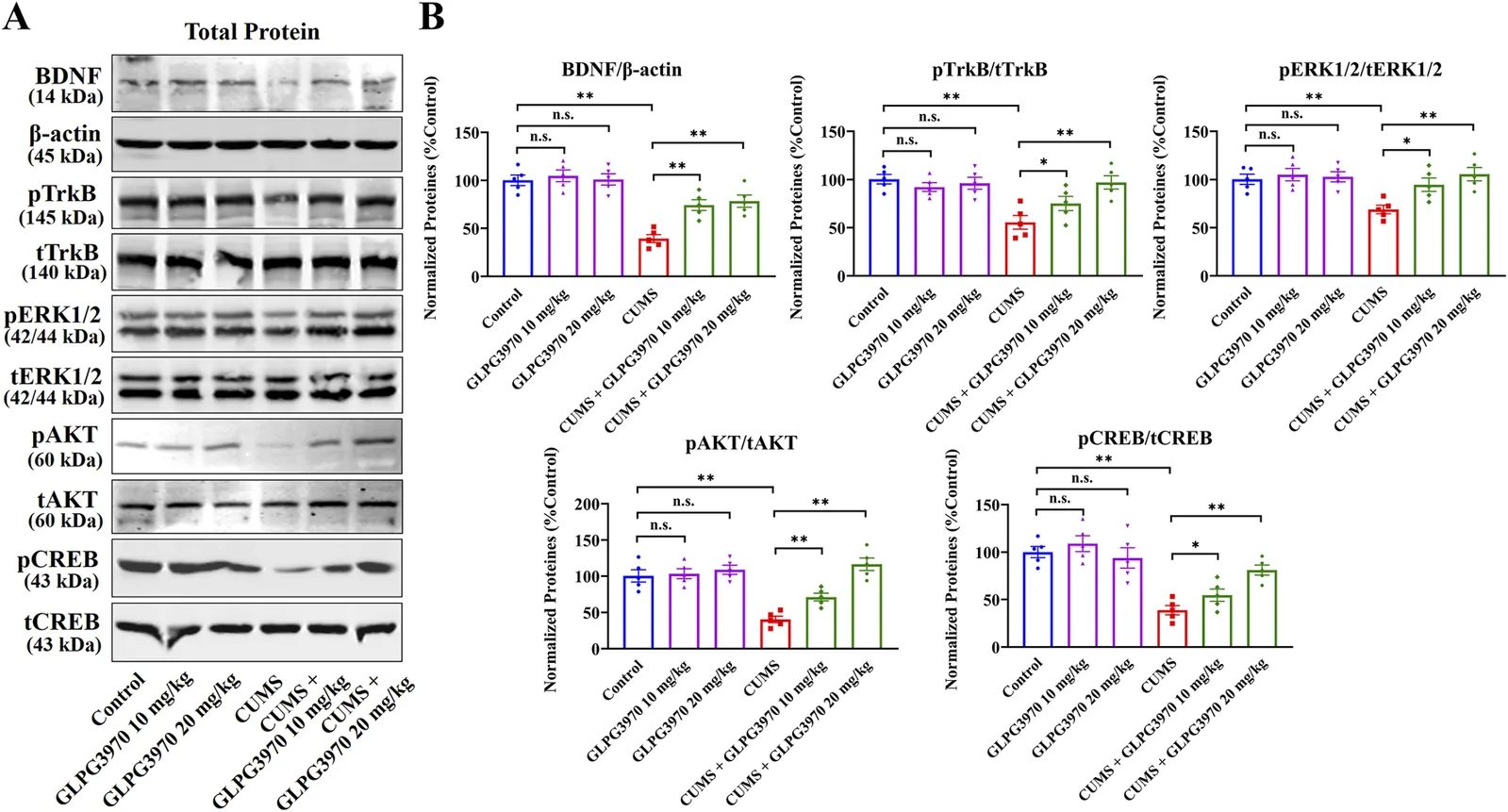
The reduction in hippocampal BDNF signaling in mice exposed to CUMS was also reversed by i.p. administration of GLPG3970. **(A)** Representative Western blot images (with molecular weight markers). **(B)** Quantitative analyses showing the effects of CUMS and GLPG3970 on the protein levels of BDNF, pTrkB, pERK1/2, pAKT, pCREB, β-actin, total TrkB, total ERK1/2, total AKT, and total CREB in the hippocampus of mice. All results are presented as means ± S.E.M. of *n* = 5 mice per group; *P < 0.05, **P < 0.01 by two-way ANOVA with Bonferroni correction; n.s., no significance.

**FIGURE 6:**
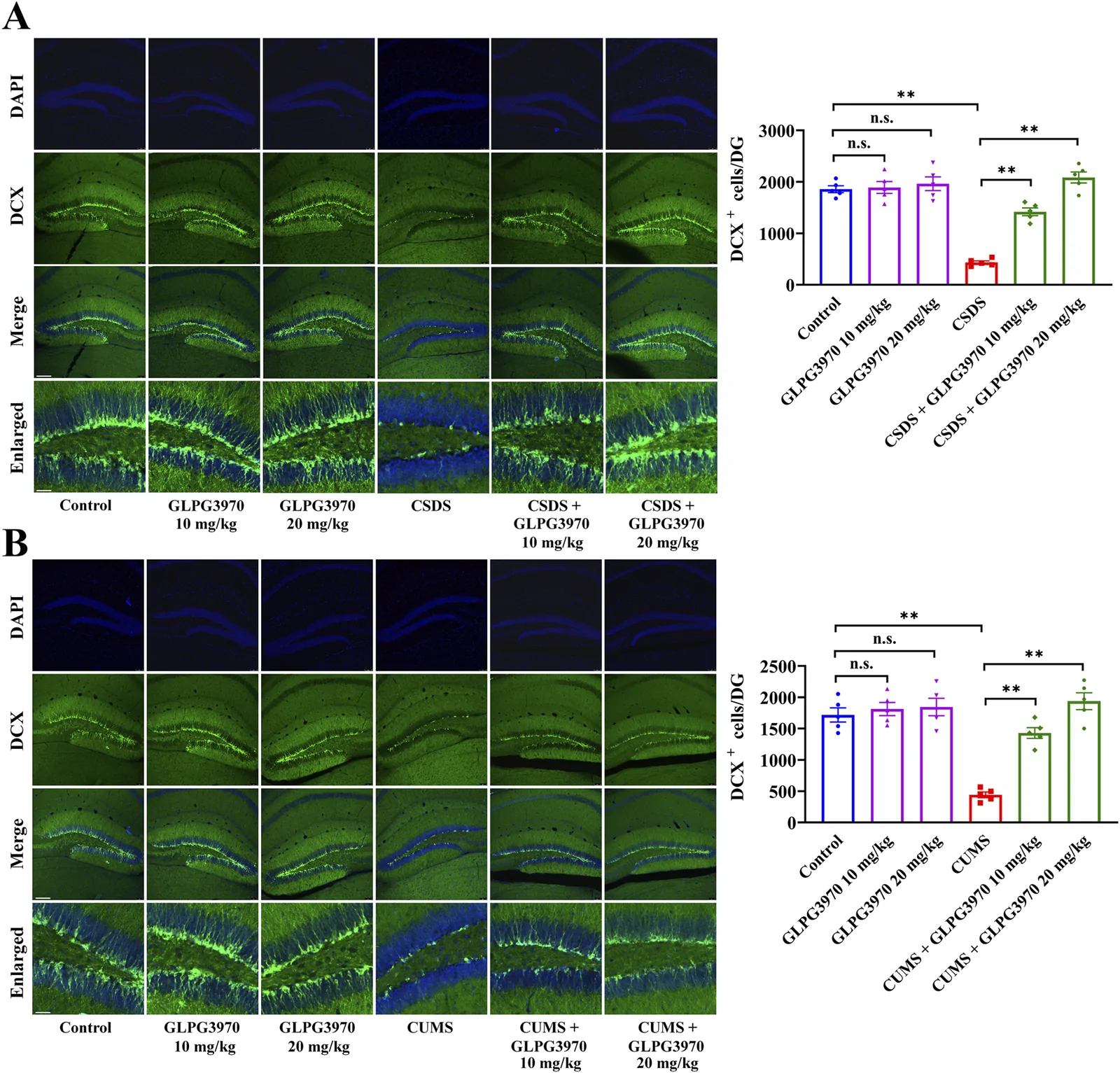
The decreased number of DCX^+^ cells in the DG of mice exposed to CSDS or CUMS was reversed by i.p. administration of GLPG3970. **(A)** Representative confocal microscopic images showing the staining of DCX (green) and DAPI (blue) in the DG of CSDS-exposed mice. Scale bars: 150 μm (overview) and 37.5 μm (enlarged). Quantitative analyses showing that GLPG3970 treatment increased the number of DCX^+^ cells in the DG of CSDS-exposed mice. **(B)** Representative confocal microscopic images showing the staining of DCX (green) and DAPI (blue) in the DG of CUMS-exposed mice. Scale bars: 150 μm (overview) and 37.5 μm (enlarged). Quantitative analyses showing that GLPG3970 treatment increased the number of DCX^+^ cells in the DG of CUMS-exposed mice. All data are presented as means ± S.E.M. of *n* = 5 mice per group; **P < 0.01 by two-way ANOVA with Bonferroni correction; n.s., no significance.

**FIGURE 7:**
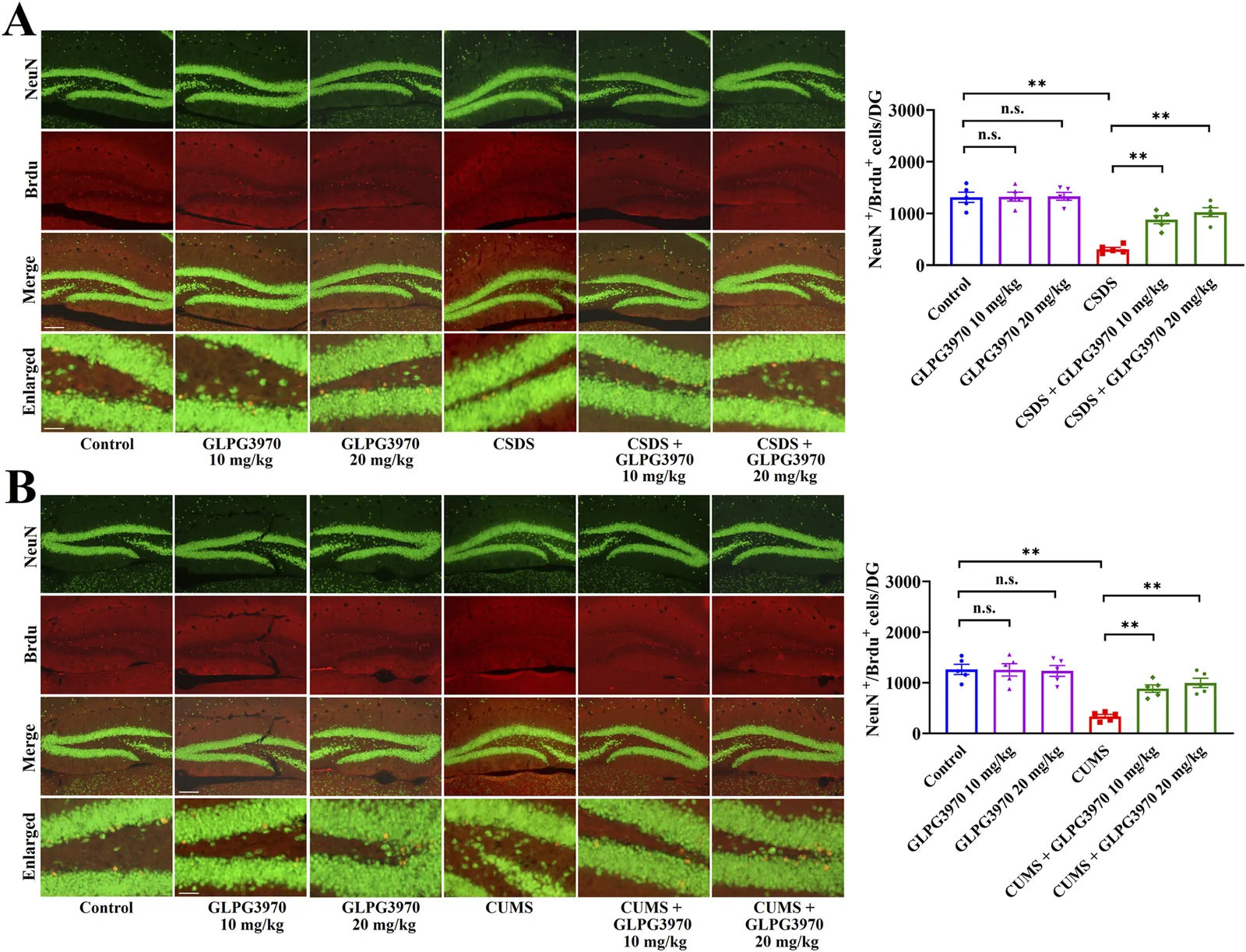
The decreased number of NeuN^+^/BrdU^+^ cells in the DG of mice exposed to CSDS or CUMS was also reversed by i.p. administration of GLPG3970. **(A)** Representative confocal microscopic images showing the staining of NeuN (green) and BrdU (Red) in the DG of CSDS-exposed mice. Scale bars: 150 μm (overview) and 37.5 μm (enlarged). Quantitative analyses showing that GLPG3970 treatment increased the number of NeuN^+^/BrdU^+^ cells in the DG of CSDS-exposed mice. **(B)** Representative confocal microscopic images showing the staining of NeuN (green) and BrdU (Red) in the DG of CUMS-exposed mice. Scale bars: 150 μm (overview) and 37.5 μm (enlarged). Quantitative analyses showing that GLPG3970 treatment increased the number of NeuN^+^/BrdU^+^ cells in the DG of CUMS-exposed mice. All data are presented as means ± S.E.M. of *n* = 5 mice per group; **P < 0.01 by two-way ANOVA with Bonferroni correction; n.s., no significance.

### 3.3 The antidepressant-like effects induced by i.p. GLPG3970 administration in mice are dependent on the regulatory mediation of hippocampal SIK2, CRTC1, CREB, and BDNF

Given that GLPG3970 intervention blocks the inhibitory effects of CSDS and CUMS on the hippocampal SIK2-CRTC1 system and BDNF signaling pathway, this study further employed AAV-mediated gene transfer (AAV-SIK2, AAV-CRTC1-shRNA, AAV-CREB-shRNA, and AAV-BDNF-shRNA) to verify whether the antidepressant-like effects of GLPG3970 depend on the regulation of hippocampal SIK2, CRTC1, CREB, and BDNF. Western blotting analysis confirmed the efficacy of AAV-SIK2 [ANOVA: F(2, 12) = 34.635, *P* < 0.01], CRTC1-shRNA [ANOVA: F(2, 12) = 28.187, *P* < 0.01], CREB-shRNA [ANOVA: F(2, 12) = 24.302, *P* < 0.01], and BDNF-shRNA [ANOVA: F(2, 12) = 26.194, *P* < 0.01] (n = 5, *P <* 0.01; Figure 8).

**FIGURE 8:**
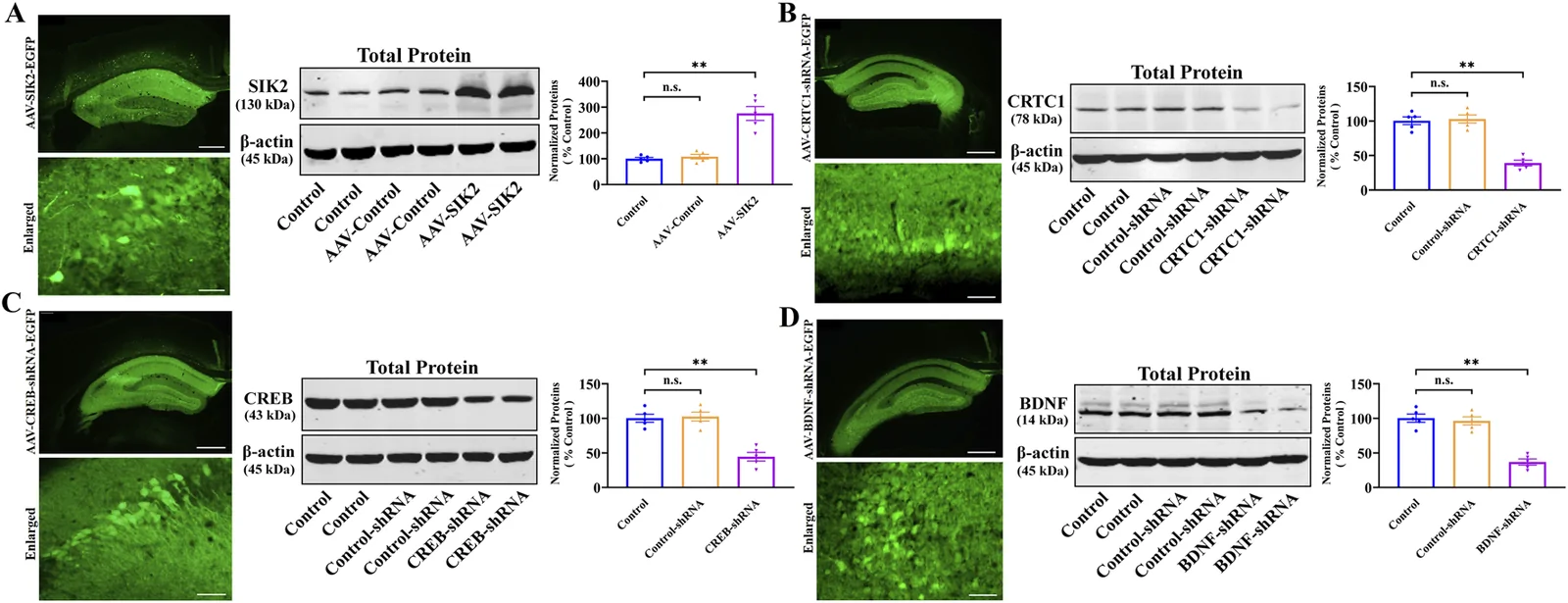
The efficacy of stereotaxic injection of AAV-SIK2-EGFP/AAV-CRTC1-shRNA-EGFP/AAV-CREB-shRNA-EGFP/AAV-BDNF-shRNA-EGFP in overexpressing SIK2 or knocking down CRTC1/CREB/BDNF expression in the hippocampus was validated by EGFP fluorescence and Western blot analysis performed 14 days after infusion. Scale bars: 400 μm (overview) and 50 μm (enlarged) apply to all fluorescence images in **(A–D)**. **(A)** Representative fluorescence images of the hippocampus and corresponding Western blot analysis confirming enhanced SIK2 expression following AAV-SIK2 infusion. **(B)** Representative fluorescence images of the hippocampus and corresponding Western blot analysis confirming reduced CRTC1 expression following AAV-CRTC1-shRNA infusion. **(C)** Representative fluorescence images of the hippocampus and corresponding Western blot analysis confirming reduced CREB expression following AAV-CREB-shRNA infusion. **(D)** Representative fluorescence images of the hippocampus and corresponding Western blot analysis confirming reduced BDNF expression following AAV-BDNF-shRNA infusion. All data are presented as means ± S.E.M. of *n* = 5 mice per group; **P < 0.01 by one-way ANOVA with Tukey’s test; n.s., no significance.

As shown in Figures 9A,B, hippocampal SIK2 overexpression significantly reduced the antidepressant-like effects of GLPG3970 (20 mg/kg) against CSDS and CUMS. Compared to both (CSDS/CUMS + GLPG3970)-treated and (CSDS/CUMS + GLPG3970 + AAV-Control)-treated groups, mice in (CSDS/CUMS + GLPG3970 + AAV-SIK2)-treated group exhibited lower sucrose preference [ANOVA for CSDS: F(6, 63) = 22.194, *P* < 0.01. ANOVA for CUMS: F(6, 63) = 25.037, *P* < 0.01] and greater immobility in the FST [ANOVA for CSDS: F(6, 63) = 13.715, *P* < 0.01. ANOVA for CUMS: F(6, 63) = 16.403, *P* < 0.01] and TST [ANOVA for CSDS: F(6, 63) = 19.661, *P* < 0.01. ANOVA for CUMS: F(6, 63) = 15.118, *P* < 0.01] (n = 10, *P* < 0.01). In addition, mice in (CSDS + GLPG3970 + AAV-SIK2)-treated group interacted less frequently with the intruder than mice in (CSDS + GLPG3970)-treated and (CSDS + GLPG3970 + AAV-Control)-treated groups [ANOVA: F(6, 63) = 37.202, *P* < 0.01] (n = 10, *P* < 0.01; Figure 9A).

**FIGURE 9:**
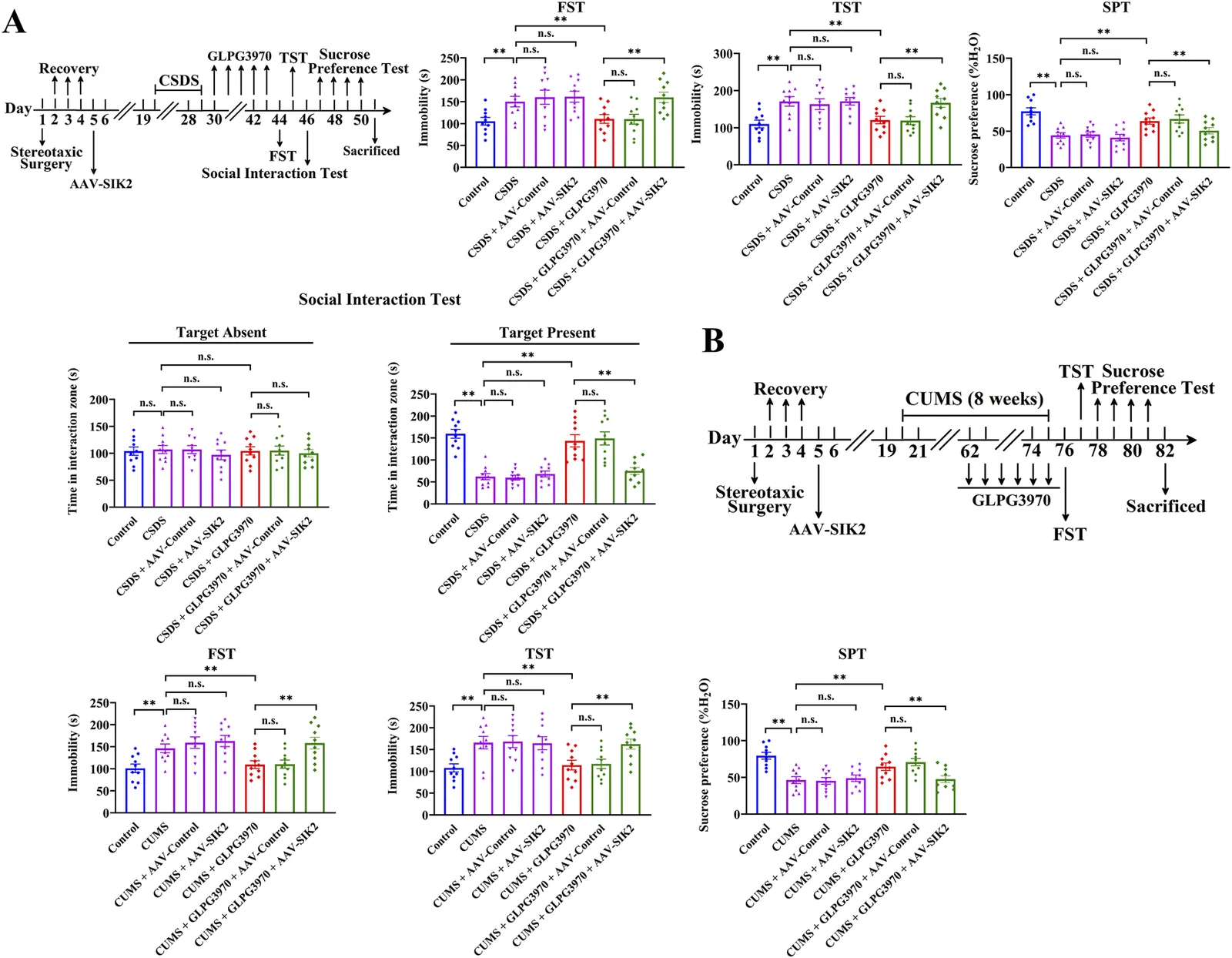
The antidepressant-like effects of GLPG3970 in mice exposed to CSDS or CUMS were attenuated by AAV-SIK2 pre-treatment. **(A)** The experimental procedure timeline and behavioral testing results in CSDS-exposed mice. Pre-treatment with AAV-SIK2 significantly blocked the reversal effects of GLPG3970 against the CSDS-induced depressive-like behaviors in the FST, TST, SPT and social interaction test. **(B)** The experimental procedure timeline and behavioral testing results in CUMS-exposed mice. Pre-treatment with AAV-SIK2 significantly blocked the reversal effects of GLPG3970 against the CUMS-induced depressive-like behaviors in the FST, TST and SPT. All results are presented as means ± S.E.M. of *n* = 10 mice per group; **P < 0.01 by one-way ANOVA with Tukey’s test; n.s., no significance.

Furthermore, compared with mice in the non-CRTC1-knockdown groups [(CSDS/CUMS + GLPG3970) and (CSDS/CUMS + GLPG3970 + Control-shRNA)], mice in the (CSDS/CUMS + GLPG3970 + CRTC1-shRNA) treatment groups exhibited depressive-like behaviors as evidenced by significantly prolonged immobility time in the FST [ANOVA for CSDS: F(6, 63) = 17.547, *P* < 0.01. ANOVA for CUMS: F(6, 63) = 18.099, *P* < 0.01] and TST [ANOVA for CSDS: F(6, 63) = 19.386, *P* < 0.01. ANOVA for CUMS: F(6, 63) = 20.514, *P* < 0.01], accompanied by a marked reduction in sucrose preference [ANOVA for CSDS: F(6, 63) = 21.774, *P* < 0.01. ANOVA for CUMS: F(6, 63) = 23.765, *P* < 0.01] (n = 10, *P <* 0.01; Figures 10A,B). Additionally, the frequency of social interaction with intruder CD1 mice was significantly decreased in the (CSDS + GLPG3970 + CRTC1-shRNA)-treated group [ANOVA: F(6, 63) = 30.102, *P* < 0.01] (n = 10, *P <* 0.01; Figure 10A). Similarly, relative to mice in the non-CREB-knockdown groups [(CSDS/CUMS + GLPG3970) and (CSDS/CUMS + GLPG3970 + Control-shRNA)], mice treated with (CSDS/CUMS + GLPG3970 + CREB-shRNA) showed significantly increased immobility time in the FST [ANOVA for CSDS: F(6, 63) = 16.495, *P* < 0.01. ANOVA for CUMS: F(6, 63) = 19.183, *P* < 0.01] and TST [ANOVA for CSDS: F(6, 63) = 18.507, *P* < 0.01. ANOVA for CUMS: F(6, 63) = 17.633, *P* < 0.01], as well as reduced sucrose preference [ANOVA for CSDS: F(6, 63) = 27.167, *P* < 0.01. ANOVA for CUMS: F(6, 63) = 25.487, *P* < 0.01] (n = 10, *P <* 0.01; Figures 11A,B), along with a lower frequency of social interaction with intruder CD1 mice [ANOVA: F(6, 63) = 33.051, *P* < 0.01] (n = 10, *P <* 0.01; Figure 11A). Consistently, relative to mice in the non-BDNF-knockdown groups [(CSDS/CUMS + GLPG3970) and (CSDS/CUMS + GLPG3970 + Control-shRNA)], mice in the (CSDS/CUMS + GLPG3970 + BDNF-shRNA) treatment group also displayed obvious depressive-like behavioral phenotypes, including prolonged immobility time in the FST [ANOVA for CSDS: F(6, 63) = 15.672, *P* < 0.01. ANOVA for CUMS: F(6, 63) = 19.023, *P* < 0.01] and TST [ANOVA for CSDS: F(6, 63) = 18.046, *P* < 0.01. ANOVA for CUMS: F(6, 63) = 16.985, *P* < 0.01], decreased sucrose preference [ANOVA for CSDS: F(6, 63) = 27.011, *P* < 0.01. ANOVA for CUMS: F(6, 63) = 24.819, *P* < 0.01] (n = 10, *P <* 0.01; Figures 12A,B), and diminished frequency of social interaction with intruder CD1 mice [ANOVA: F(6, 63) = 34.655, *P* < 0.01] (n = 10, *P <* 0.01; Figure 12A).

**FIGURE 10:**
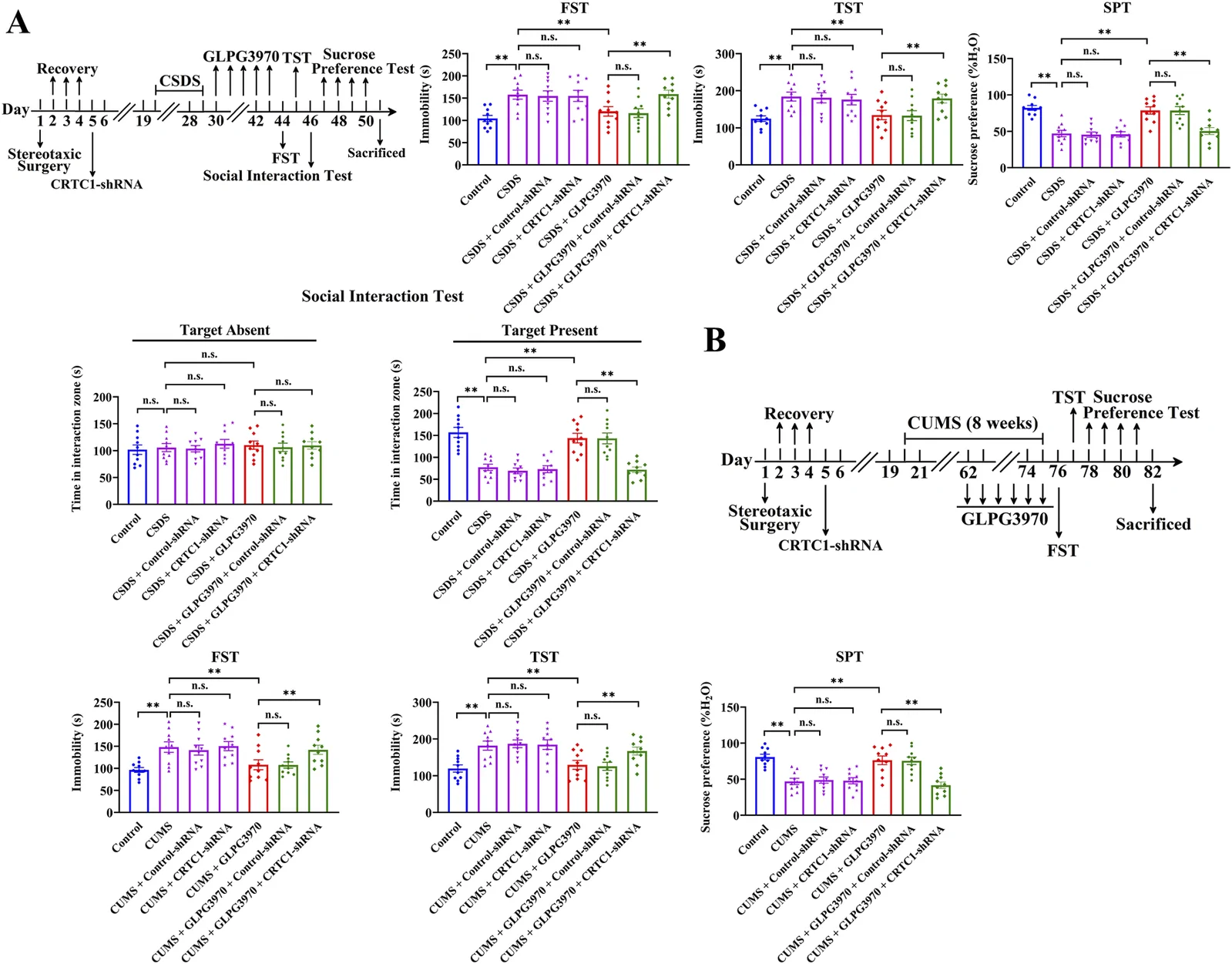
The antidepressant-like effects of GLPG3970 in mice exposed to CSDS or CUMS were abolished by AAV-CRTC1-shRNA pre-treatment. **(A)** The experimental procedure timeline and behavioral testing results in CSDS-exposed mice. Pre-treatment with AAV-CRTC1-shRNA significantly blocked the reversal effects of GLPG3970 against the CSDS-induced depressive-like behaviors in the FST, TST, SPT and social interaction test. **(B)** The experimental procedure timeline and behavioral testing results in CUMS-exposed mice. Pre-treatment with AAV-CRTC1-shRNA significantly blocked the reversal effects of GLPG3970 against the CUMS-induced depressive-like behaviors in the FST, TST and SPT. All results are presented as means ± S.E.M. of *n* = 10 mice per group; **P < 0.01 by one-way ANOVA with Tukey’s test; n.s., no significance.

**FIGURE 11:**
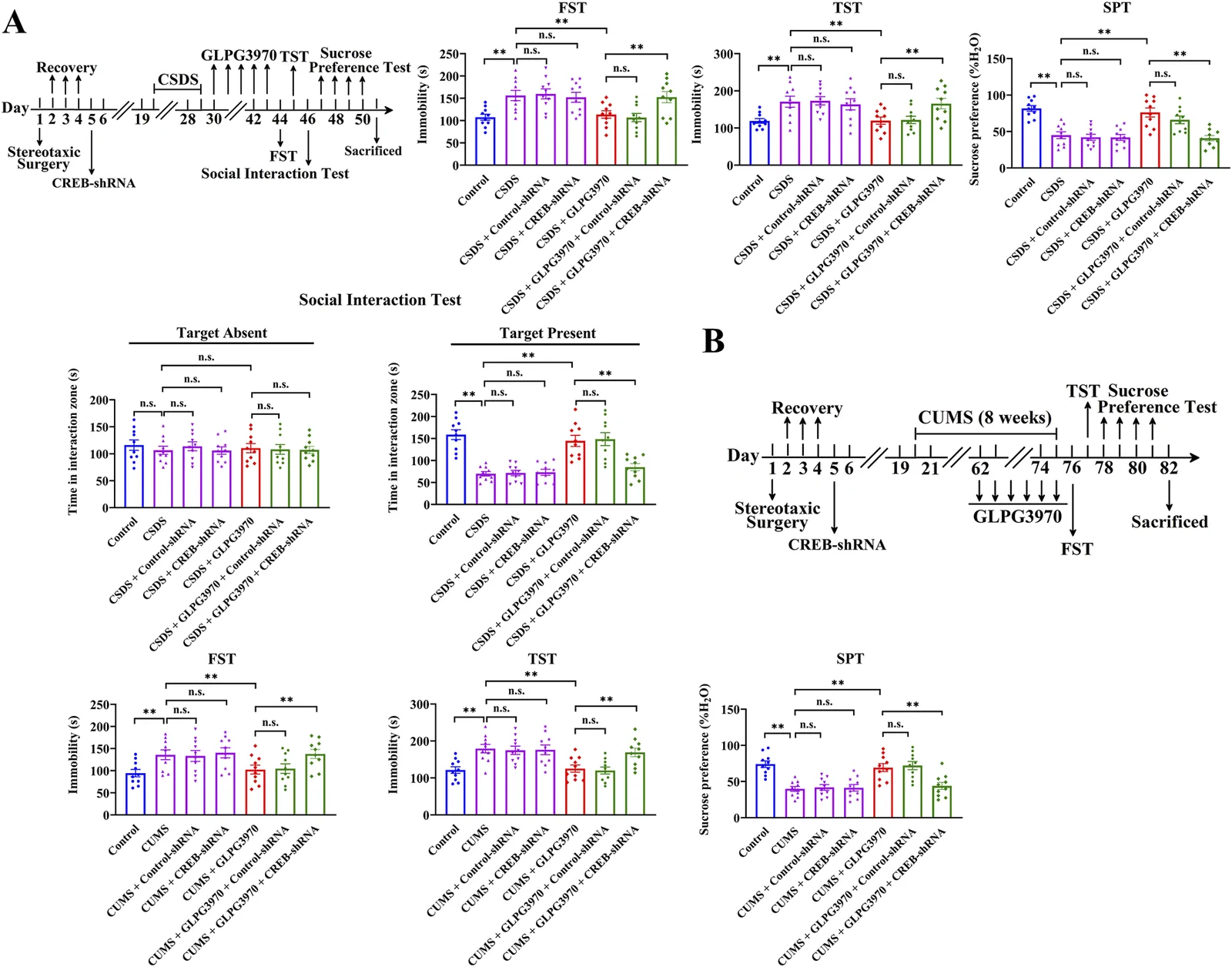
The antidepressant-like effects of GLPG3970 in mice exposed to CSDS or CUMS were abrogated by AAV-CREB-shRNA. **(A)** The experimental procedure timeline and behavioral testing results in CSDS-exposed mice. Pre-treatment with AAV-CREB-shRNA significantly blocked the reversal effects of GLPG3970 against the CSDS-induced depressive-like behaviors in the FST, TST, SPT and social interaction test. **(B)** The experimental procedure timeline and behavioral testing results in CUMS-exposed mice. Pre-treatment with AAV-CREB-shRNA significantly blocked the reversal effects of GLPG3970 against the CUMS-induced depressive-like behaviors in the FST, TST and SPT. All results are presented as means ± S.E.M. of *n* = 10 mice per group; **P < 0.01 by one-way ANOVA with Tukey’s test; n.s., no significance.

**FIGURE 12:**
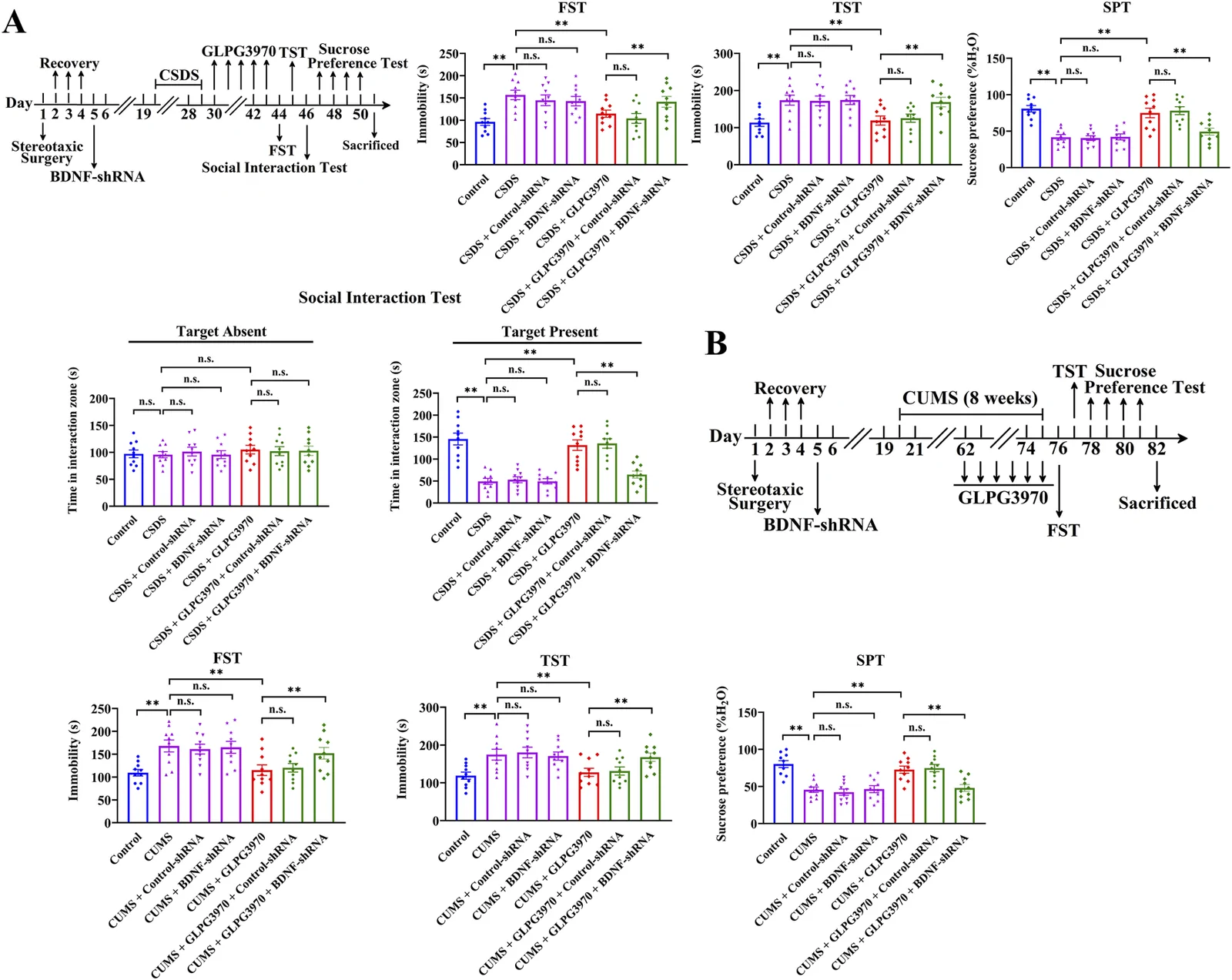
The antidepressant-like effects of GLPG3970 in mice exposed to CSDS or CUMS were eliminated by AAV-BDNF-shRNA. **(A)** The experimental procedure timeline and behavioral testing results in CSDS-exposed mice. Pre-treatment with AAV-BDNF-shRNA significantly blocked the reversal effects of GLPG3970 against the CSDS-induced depressive-like behaviors in the FST, TST, SPT and social interaction test. **(B)** The experimental procedure timeline and behavioral testing results in CUMS-exposed mice. Pre-treatment with AAV-BDNF-shRNA significantly blocked the reversal effects of GLPG3970 against the CUMS-induced depressive-like behaviors in the FST, TST and SPT. All results are presented as means ± S.E.M. of *n* = 10 mice per group; **P < 0.01 by one-way ANOVA with Tukey’s test; n.s., no significance.

Collectively, these gene knockdown experiments confirm that the hippocampal SIK2-CRTC1-CREB-BDNF signaling pathway is essential for mediating the antidepressant-like effects of GLPG3970 in mice.

## 4 Discussion

Depression is a common chronic mental disorder with complex pathogenesis involving genetics, environment, neurotransmitter imbalance, and impaired neuroplasticity (Dean and Keshavan, 2017; Krishnan and Nestler, 2008; McEwen and Gianaros, 2010; Ménard et al., 2016). Current antidepressant drug development exhibits multi-directional exploration. Monoamine-based antidepressants focus on multidimensional neurotransmitter regulation, as exemplified by multi-target monoamine reuptake inhibition (e.g., toludesvenlafaxine), precise modulation of monoamine receptors (e.g., SEP-363856), and monoamine transporter–protein coupled dissociation (e.g., ZZL-7) (Li et al., 2024; Sun et al., 2022; Vasiliu, 2023). Although these novel monoaminergic agents demonstrate advantages in certain therapeutic metrics, they have failed to overcome the core limitations of traditional drugs such as SSRIs and SNRIs. In contrast, non-monoaminergic antidepressants have emerged as a prevailing direction in antidepressant development, and these agents regulate synaptic plasticity, neurogenesis, neuroinflammation, and neuroendocrine pathways (Dean and Keshavan, 2017; Flores et al., 2022; Massart et al., 2012; Pariante and Lightman, 2008; Pastis et al., 2023).

Previous studies have demonstrated that hippocampal SIK2 is regulated by chronic stress (Jiang et al., 2019). Based on this, the present study aimed to investigate the antidepressant-like effects of GLPG3970, a newly-developed selective SIK2/3 inhibitor. Our results showed that repeated administration of GLPG3970 normalized hippocampal SIK2-CRTC1-CREB-BDNF signaling and counteracted chronic stress-induced depressive-like behaviors in mice. These findings align with previous studies on other SIK inhibitors (ARN-3236 and HG-9-91–01) that also effectively alleviated depressive-like behavioral phenotypes in rodents (Huang et al., 2023; Liu et al., 2020). The convergence of these structurally distinct SIK inhibitors on the CRTC1-CREB-BDNF pathway reinforces the robustness of SIK2 as a valid antidepressant target. However, the novelty of the present study lies in a critical distinction that differentiates GLPG3970 from these prior tool compounds: GLPG3970 is a clinical-stage compound that has successfully completed Phase I clinical trials with established human safety, tolerability, and pharmacokinetic profiles (Peixoto et al., 2024; van der Plas et al., 2025). Unlike ARN-3236 and HG-9-91–01, which are laboratory tool compounds lacking systematic drug metabolism and pharmacokinetic optimization and human safety data, GLPG3970 possesses favorable oral bioavailability and drug-like properties that are directly translatable to clinical development. This positions GLPG3970 not merely as a probe for target validation but as a viable candidate for drug repurposing in neuropsychiatric indications, offering a direct bridge from preclinical efficacy demonstration to potential clinical application. Moreover, GLPG3970 showed comparable efficacy to fluoxetine at an equivalent dose (20 mg/kg), suggesting that this compound may offer an alternative for patients with poor responses to monoaminergic antidepressants, consistent with the paradigm shift toward non-monoaminergic targets (Cui et al., 2024).

GLPG3970 joins a growing arsenal of non-monoaminergic antidepressants that target distinct pathophysiological mechanisms. The glutamatergic modulator ketamine and its enantiomer esketamine have garnered considerable attention for their rapid and sustained antidepressant effects, which are mediated in part through activation of mammalian target of rapamycin (mTOR) and subsequent increases in synaptic protein synthesis (Qin et al., 2024; Zanos and Gould, 2018). GLPG3970 engages the SIK2-CRTC1-CREB-BDNF pathway, promoting neurotrophic signaling and hippocampal neurogenesis through a mechanism that does not directly involve the glutamatergic system. Interestingly, BDNF itself can activate mTOR through the TrkB-PI3K-AKT pathway (Qin et al., 2024), suggesting that these two approaches may converge on shared downstream neuroplasticity-enhancing pathways. Similarly, other neurotrophin-based strategies, including small-molecule TrkB agonists and BDNF mimetics, have been explored as potential antidepressants (Fukuda et al., 2016; Gudasheva et al., 2019). However, GLPG3970 acts upstream of BDNF by relieving the chronic stress-induced suppression of CREB-mediated BDNF transcription, representing a distinct regulatory node in the neurotrophic cascade. Neuroimmune modulation has also emerged as a promising antidepressant target, particularly for patients with elevated inflammatory markers (Nava et al., 2025). Interestingly, Peixoto et al. (2024) demonstrated that GLPG3970 modulates the secretion of both pro-inflammatory (TNFα) and anti-inflammatory (IL-10) cytokines in immune cells, suggesting that its antidepressant-like effects may involve, in part, attenuation of neuroinflammation, a mechanism shared with other immunomodulatory agents being investigated for depression, such as minocycline and infliximab (Peixoto et al., 2024).

It is noteworthy that, unlike in the hippocampus of stressed mice, no discernible changes in SIK2 expression were observed in the non-stressed control group. This finding suggests that the observed alterations in SIK2 expression levels may result from pharmacological inhibition of its kinase activity rather than from transcriptional regulation or post-translational modifications. We hypothesize that this phenomenon may be mechanistically linked to BDNF production and subsequent feedback regulation. In addition to the well-known downstream ERK1/2 and AKT pathways, several previous studies have indicated that BDNF also activates CaMKIV (Espasandín et al., 2022; Spencer et al., 2008; Wu et al., 2022). Furthermore, Sasaki et al. demonstrated that CaMKIV promotes proteolytic degradation of SIK2 in cortical neurons by mediating phosphorylation at the Thr484 residue of SIK2 (Sasaki et al., 2011). Based on this evidence, we hypothesize that GLPG3970 may facilitate SIK2 degradation via activation of the BDNF-CaMKIV pathway, thereby potentiating its inhibitory effect on SIK2. This hypothesis will be tested in future studies through experiments specifically investigating CaMKIV.

Our genetic knockout experiments have confirmed that GLPG3970 exerts antidepressant-like effects via the hippocampal SIK2-CRTC1-CREB-BDNF pathway. In the present study, BDNF was selected as a key downstream molecular target mediating the protective effects of GLPG3970 against chronic stress, primarily for two reasons: first, BDNF is closely implicated in the pathogenesis of depression; and second, BDNF biosynthesis is tightly regulated by CRTC1 and CREB. Notably, the regulatory network of BDNF is not restricted to the field of depression; as a pivotal regulator of adult hippocampal neurogenesis, dysregulation of BDNF also contributes to the pathological progression of multiple neurological disorders, including Parkinson’s disease, Alzheimer’s disease, and Huntington’s disease (Ali et al., 2024; Azman and Zakaria, 2025; Gao et al., 2022). Given these associations, evaluating the potential therapeutic efficacy of GLPG3970 for central nervous system (CNS) disorders beyond depression represents a critical direction for future research. It should be emphasized, however, that the neurobiological pathogenesis of depression is extremely complex, and its regulatory network extends far beyond the single molecule BDNF. Accumulating evidence has confirmed that CREB, as a core transcription factor, can regulate a range of downstream effector molecules, including mTOR, vascular endothelial growth factor (VEGF), and peroxisome proliferator-activated receptor α (PPARα), all of which have been verified to be involved in the pathological regulation of depression (Cheng et al., 2025; Moya-Alvarado et al., 2023; Roy et al., 2013). Meanwhile, the SIK family exerts pleiotropic regulatory functions; in addition to its canonical role in regulating the CRTC family, SIKs can also target the polarity protein Par3, the Hippo signaling pathway, and cytoplasmic histone deacetylase 4 (HDAC4), thereby participating in multiple physiological and pathological processes such as cell proliferation, differentiation, and stress responses (Shi et al., 2025; Vanlandewijck et al., 2018; Wehr et al., 2013). Additionally, Peixoto et al. demonstrated that GLPG3970 can modulate the secretion of several inflammation-related cytokines, including the pro-inflammatory cytokine TNFα and the anti-inflammatory cytokine IL-10, and cytokine imbalance also plays a pivotal role in the pathophysiological progression of depression (Lotrich, 2015; Peixoto et al., 2024). Collectively, these findings suggest that the antidepressant-like effects of GLPG3970 may not depend on a single target but rather be achieved through the synergistic regulation of multiple signaling pathways, among which the SIK2-CRTC1-CREB-BDNF pathway constitutes one of the core regulatory axes. To further clarify the multi-target regulatory mechanism of GLPG3970, our next study will adopt a variety of shRNA-specific knockdown strategies to systematically dissect the synergistic and independent roles of each target in mediating the antidepressant-like effects of GLPG3970.

The present study has several limitations. First, although we demonstrated that GLPG3970 modulates hippocampal SIK2-CRTC1 signaling, direct measurement of brain drug concentrations and SIK2 kinase activity would provide stronger evidence for target engagement. We acknowledge that dedicated brain pharmacokinetic studies and *in vitro* kinase activity assays will be important to confirm central target engagement. Second, only male C57BL/6J mice were used, while female subjects were not included. Given the pronounced sexual dimorphism in the pathogenesis of depression, the generalizability of the present findings across sexes is limited. Third, only two rodent models of depression were employed, which cannot fully recapitulate the complex behavioral phenotypes and pathological mechanisms of human depression. Fourth, while our study focused on depressive-like behaviors, depression is often comorbid with anxiety and cognitive deficits. The lack of behavioral assessments for anxiety-like behaviors (e.g., elevated plus maze, novelty-suppressed feeding) and cognitive functions (e.g., novel object recognition) represents a limitation of the present study. Fifth, the relatively short treatment duration (2 weeks) and the lack of follow-up studies to evaluate the persistence of behavioral and molecular effects after drug discontinuation limit the translational relevance of our findings. Chronic treatment regimens and drug-withdrawal paradigms in future studies will be valuable to assess whether GLPG3970 produces sustained antidepressant-like effects and whether it offers advantages over existing antidepressants in preventing relapse. Sixth, although our Western blot and Co-IP data demonstrated GLPG3970-induced changes in CRTC1 nuclear/cytoplasmic distribution and CRTC1-CREB binding, immunofluorescence visualization of CRTC1 subcellular localization in hippocampal neurons would provide complementary visual evidence. These limitations will be addressed in future studies.

In addition to exerting antidepressant-like effects by inhibiting SIK2, GLPG3970 also inhibits SIK1, which possesses antidepressant-like properties in the paraventricular nucleus (PVN) of the hypothalamus (Wang et al., 2024). This off-target inhibition may partially counteract its antidepressant-like effects in the hippocampus. However, the inhibitory potency of GLPG3970 against SIK1 (IC50 = 282.8 nM) is considerably weaker than that against SIK2 (IC50 = 7.8 nM). Therefore, despite the potential adverse effect of SIK1 inhibition, the more potent inhibition of SIK2 by GLPG3970 results in an overall antidepressant-like phenotype.

In summary, the present study systematically demonstrates that GLPG3970 exerts antidepressant-like effects by inhibiting hippocampal SIK2 and regulating the CRTC1-CREB-BDNF signaling pathway. These findings not only deepen and expand the understanding of the pharmacological mechanisms underlying GLPG3970 but also further identify hippocampal SIK2 as a potential novel target for antidepressant drug development.

## Data Availability

The original contributions presented in the study are included in the article/Supplementary Material, further inquiries can be directed to the corresponding authors.

## Supplementary material

SUPPLEMENTARY FIGURE S1 CSDS exposure **(A)**, CUMS exposure **(B)**, i.p. injection of fluoxetine and GLPG3970 all did not affect the locomotor activity of mice, as revealed by the open field test (OFT). No significant differences were found among all groups in the number of squares that a mouse crossed in the central or peripheral area. All data are presented as means ± S.E.M. of n = 10 mice per group; n.s., no significance (Two-way ANOVA + Bonferroni correction).
