# Supplementary material for: The selective SIK2/3 inhibitor GLPG3970 exhibits antidepressant-like effects via the hippocampal SIK2-CRTC1-CREB-BDNF pathway in chronic stress models
Source: Front Pharmacol. 2026 Oct 1;17:1895911. doi: 10.3389/fphar.2026.1895911 (PMC13668743; doi:10.3389/fphar.2026.1895911)
Supplement: Supplementary file 1 [file Supplementaryfile1.docx]

The protein marker used in this study was provided by Beyotime Biotechnology (No. P0068).

These are the original uncropped images of western blots for Figure 1B.

These are the original uncropped images of western blots for Figure 2B.

These are the original uncropped images of western blots for Figure 3A.

These are the original uncropped images of western blots for Figure 3B.

These are the original uncropped images of western blots for Figure 4A.

These are the original uncropped images of western blots for Figure 5A.

These are the original uncropped images of western blots for Figure 8.
